# Measuring quality of life in advanced chronic liver disease with FACT-Hep: Findings from the PAL LIVER trial

**DOI:** 10.1097/HC9.0000000000001043

**Published:** 2026-08-28

**Authors:** Manisha Verma, Binu V. John, Don Rockey, Sid Barritt, Victor Navarro, Linda K. Shaw, Andrzej Kosinski, Elliot B. Tapper, Archita Desai

**Affiliations:** 1 Department of Medicine, Jefferson Einstein Hospital, Thomas Jefferson University, Philadelphia, Pennsylvania, USA; 2 Department of Medicine, University of Miami and Miami VA Health System, Miami, Florida, USA; 3 Department of Medicine, Medical University of South Carolina, Charleston, South Carolina, USA; 4 Department of Medicine, University of North Carolina, Chapel Hill, North Carolina, USA; 5 Duke Clinical Research Institute, Duke University, Durham, North Carolina, USA; 6 Division of Gastroenterology and Hepatology, University of Michigan, Ann Arbor, Michigan, USA; 7 Department of Medicine, Indiana University, Indianapolis, Indiana, USA

**Keywords:** decompensated cirrhosis, disease burden, quality of life, symptom burden

## Abstract

**Background:**

Patient-reported outcome measures (PROMs) are increasingly incorporated into hepatology research, yet few have been rigorously evaluated, particularly among patients with decompensated cirrhosis. The Functional Assessment of Cancer Therapy–Hepatobiliary (FACT-Hep) captures multidimensional liver disease–specific burden, while the disease-agnostic PROMIS-29 assesses physical and mental health broadly. We evaluated the performance of FACT-Hep and its relationship to PROMIS-29 among participants with cirrhosis or hepatocellular carcinoma (HCC) enrolled in a prospective multicenter clinical trial.

**Methods:**

Baseline data were analyzed from 935 participants enrolled in the PAL LIVER trial. This study enrolled patients with Child–Turcotte–Pugh (CTP) B/C cirrhosis and/or with HCC. Health-related quality of life (HRQoL) was assessed using FACT-Hep and PROMIS-29. Correlations and multivariable linear regression models examined associations between PROM scores and clinical characteristics.

**Results:**

Among 935 patients, the mean FACT-Hep total score was 116 (SD 28.16), with worse scores among women, young participants (age <50 y), and Whites (all *p*<0.05). Patients with CTP B/C cirrhosis (83%) had worse HRQoL than those with HCC alone (28%) (*p*<0.0001). Younger age, higher MELD 3.0, smoking, and worse ECOG status were independently associated with total FACT-Hep score. FACT-Hep correlated with PROMIS preference scores (correlation coefficient=0.78). MELD 3.0 demonstrated a stronger association with total FACT-Hep score than with PROMIS measures.

**Conclusions:**

HRQoL measures provide important information distinct from disease severity indices. HRQoL impairment in patients with advanced chronic liver disease is driven primarily by decompensation, particularly ascites and hepatic encephalopathy. FACT-Hep and PROMIS-29 correlate well. FACT-Hep is a valid measure and effectively captures the multidimensional liver disease–specific burden. PROMIS-29 measures broader aspects that may be influenced by many factors beyond liver disease.

## INTRODUCTION

Patients with advanced chronic liver diseases (ACLD) experience substantially impaired health-related quality of life (HRQoL), driven by a high symptom burden and complications such as ascites, hepatic encephalopathy (HE), and sarcopenia, which collectively contribute to functional decline.^1^,^2^,^3^ In this context, patient-reported outcome measures (PROMs) have emerged as essential tools in both clinical care and research, enabling a more patient-centered assessment of disease and treatment effects.^4^,^5^ Disease severity metrics such as Model for End-Stage Liver Disease (MELD) 3.0 scores are prognostic and objectively facilitate organ allocation,^6^ but they neither capture all risk nor reflect prevalent deficits in physical, emotional, and social function.^7^,^8^ PROMs that assess HRQoL, therefore provide an important independent dimension for assessing patient-centered health outcomes.^9^,^10^,^11^

The PROMIS-29 profile has gained traction in hepatology due to its brevity and ability to assess and benchmark multiple HRQoL domains relevant to patients with chronic liver disease.^12^,^13^ On the other hand, several liver disease–specific instruments are available, including the Chronic Liver Disease Questionnaire (CLDQ) and Liver Disease Quality of Life (LDQOL).^14^ While these have been validated in cohorts with chronic liver disease and compensated cirrhosis, their applicability to patients with decompensated cirrhosis remains limited.^8^ Finally, the Functional Assessment of Cancer Therapy–Hepatobiliary (FACT-Hep) is a disease-specific PROM that has been used in several clinical trials to assess treatment response and disease progression in hepatocellular cancer (HCC).^15^,^16^,^17^ The FACT-Hep HRQoL instrument was initially validated in patients with hepatobiliary cancers, including HCC, and incorporates a hepatobiliary symptom subscale intended to assess disease-specific symptoms and treatment-related concerns. Because many of these symptoms overlap with those experienced by patients with advanced chronic liver disease, FACT-Hep may have utility beyond oncology populations, although its performance in this setting has been less well studied.^18^

Comparing FACT-Hep with PROMIS-29 provides an opportunity to evaluate construct validity and domain concordance and assess whether FACT-Hep captures unique liver-specific symptomatology beyond generic measures for the ACLD population. Leveraging the well-characterized and diverse cohort of individuals with ACLD (ie, decompensated cirrhosis and HCC) from the PAL LIVER study, we aimed to (1) characterize the distribution of HRQoL scores as measured by FACT-Hep and PROMIS-29, (2) identify clinical and demographic predictors of HRQoL, and (3) evaluate the correlation of FACT-Hep and PROMIS-29. By addressing a gap in HRQoL measurement science for ACLD, this study provides essential baseline HRQoL data for ACLD and informs the design and interpretation of future trials using PROMs.

## METHODS

### Study cohort

This study used baseline data from a multisite cluster–randomized comparative effectiveness trial comparing 2 models of palliative care delivery (PAL LIVER trial, NCT03540771). The protocol and primary results have been previously described in detail.^19^ Briefly, this cluster-randomized comparative effectiveness trial compared palliative care (PC) delivered by PC specialists to trained hepatologists, by randomizing 19 US academic centers [including Veterans Administration (VA) and non-VA centers].^20^ Eligible participants included adults with decompensated cirrhosis (with a complication within the past 6 mo), and/or HCC, excluding those with Barcelona Clinic Liver Cancer (BCLC) stage D with a projected life expectancy of more than 3 months (based on clinical judgment of their hepatologist). All research was conducted in accordance with both the Declarations of Helsinki and Istanbul. Institutional review board approval was obtained at each participating site, and the data coordinating center. All participants provided informed consent prior to enrollment. Patients were enrolled from outpatient hepatology practices. After consent, participants completed FACT-Hep and PROMIS-29. Additional demographic, clinical, and laboratory data were collected via self-report and electronic health record abstraction at the time of enrollment.

### Study instruments

The Functional Assessment of Cancer Therapy–Hepatobiliary (FACT-Hep) is a 45-item, self-reported instrument comprising the 27-item FACT-General (FACT-G) and an 18-item hepatobiliary cancer subscale (HCS). FACT-G evaluates general aspects of well-being: physical well-being (PWB), social/family well-being (SWB), emotional well-being (EWB), and functional well-being (FWB). The HCS assesses liver disease–specific symptoms, including jaundice, anorexia, weight loss, and abdominal pain.^18^ Each item is rated on a 5-point Likert scale ranging from 0 (not at all) to 4 (very much). Composite scores include FACT-G (range = 0–108), Trial Outcome Index (TOI) that incorporates PWB, FWB, and HCS (range = 0–128), and overall FACT-Hep total score, calculated by combining the FACT-G and HCS (range = 0–180).^16^ Higher scores reflect better HRQoL. The recommended minimal clinically important difference (MCID) for FACT-Hep total score is 8–9 points and for FACT-Hep subscales: 2–3; FACT-G: 6–7; HCS: 5–6, and TOI: 7–8 points, respectively.^21^

The PROMIS-29 v2.0 assesses 7 health domains (each measured by 4 items) along with a single standalone item evaluating pain intensity. The domains include physical function, anxiety, depression, fatigue, sleep disturbance, ability to participate in social roles and activities, and pain interference. Domain scores (except pain intensity) are reported as a theta (T) score standardized to the 2000 US general population, with a mean of 50 (SD 10, range 0–100).^22^,^23^,^24^ Higher scores reflect more of a given domain (eg, a higher fatigue score means higher fatigue, a higher physical function score means better physical function). The recommended MCID is 3–4 points for individual PROMIS domains and 4–6 points for the summary scores.^12^ Furthermore, a preference-based health utility index, the PROMIS-Preference (PROPr) score, is derived from 7 domains: cognitive function, depression, fatigue, pain interference, physical function, sleep disturbance, and ability to participate in social roles and activities. As cognitive function was not directly assessed in this study, this domain was estimated using a previously validated linear regression model.^25^ Higher PROPr score reflects better HRQoL.

### Statistical methods

Descriptive statistics were used to summarize baseline demographics, clinical characteristics, disease severity, and HRQoL scores. Categorical variables are presented as frequencies and percentages, while continuous variables are presented as means with standard deviation or medians with ranges due to non-normal distributions.

Ceiling and floor effects were assessed for both HRQoL instruments to evaluate measurement sensitivity across the full score range. For Likert-based domain scores, ceiling or floor effects were defined as ≥15% of responses at the highest or lowest possible value (ie, 7 or 1, respectively), consistent with established criteria.^26^ For instruments scaled from 0 to 100 (eg, PROMIS-29), ceiling or floor effects were defined as ≥15% of participants scoring within the upper (90–100) or lower (0–10) decile. Spearman correlations were examined between FACT-Hep subscales and PROMIS-29 domains to assess convergent validity. Differences in HRQoL scores across categorical variables were evaluated using the Kruskal–Wallis test.

Multivariable linear regression was performed to identify predictors of FACT-Hep total score. Variable selection was performed using a combination of backward elimination and stepwise selection. Candidate predictors included: age, sex, race, ethnicity, BMI, employment status, insurance status, smoking history, primary liver disease etiology, Charlson Comorbidity Index, presence of ascites, HE, HCC, and MELD 3.0 score. We found that ECOG status was strongly correlated with all PROM scores; therefore, multivariable models without ECOG status were also considered (details in supplement: model summary, https://links.lww.com/HC9/C477).

No imputation was performed for missing data. All analyses were conducted using SAS version 9.4 (SAS Institute Inc., Cary, NC). Statistical significance was defined as a two-sided *p*-value <0.05.

## RESULTS

### Characteristics of the study population

A total of 935 patients were enrolled (Table 1). The majority (71%) were male with a median age of 64 years. Most participants were White (79%). Nearly half (48%) had Medicare, and 22% had Medicaid coverage. At enrollment, high school degree or less was noted in 49%, 14% were employed, 44% were retired, and 23% received disability benefits. Alcohol-associated liver disease (ALD) was the most common etiology (39%), followed by metabolic dysfunction–associated steatohepatitis (MASH, 28%) and viral hepatitis B or C (24%). HCC was present in 28%; among these patients, 40% had BCLC stage B and 37% BCLC stage C disease; 59% had multifocal tumors. Ascites was the most common liver-related complication (66%), and 45% had a history of hepatic encephalopathy (HE). The median MELD 3.0 score was 14, and 17% of the cohort were CTP class A, 58% class B, and 25% class C.

**TABLE 1:** Characteristics of the study cohort at baseline

Characteristic	All patients (N=935)
Female	275 (29.5%)
Age median (min–max) [N]	64.0 (21–87) [933]
Race	
White	736 (79.1%)
African American	144 (15.5%)
Hispanic or Latino	130 (14.1%)
Insurance status	
Private/HMO	297 (31.8%)
Medicare	453 (48.4%)
Medicaid^a^	274 (29.3%)
VA benefit insurance	284 (30.4%)
No/other insurance	75 (8.0%)
Education	
High school diploma or less	453 (48.7%)
Some college or more	478 (51.3%)
Marital status	
Single	227 (24.3%)
Married	466 (49.9%)
Divorced	172 (18.4%)
Widowed	68 (7.3%)
Employment status	
Not employed	179 (19.2%)
Employed	130 (13.9%)
Retired	411 (44.1%)
On disability	213 (22.8%)
Smoking status	
Never smoked	325 (34.8%)
Ex-smoker	387 (41.5%)
Current smoker	221 (23.7%)
BMI, median (min–max) [N]	28.3 (5.6–72.3) [872]
CCI (without liver disease), median (min–max) [N]	2.0 (0.0–12.0) [739]
Primary liver diagnosis	
Alcohol-related	322 (38.7%)
MASH/MAFLD	230 (27.6%)
Viral (HBV/HCB)	196 (23.6%)
Autoimmune	26 (3.1%)
Other	58 (7.0%)
MELD 3.0, median (min–max) [N]	14.1 (6.0-36.1) [787]
CTP score, median (min–max) [N]^b^	8.0 (5.0-15.0) [823]
Hepatocellular cancer^c^	263 (28.1%)
History of hepatic encephalopathy (within prior 6 mo)	
None	455 (55.3%)
Grades 1–2	309 (37.5%)
Grades 3–4	59 (7.2%)
History of ascites (within prior 6 mo)	
None	282 (34.3%)
Mild/mod (diuretic responsive)	381 (46.3%)
Severe (diuretic refractory)	160 (19.4%)
ECOG status	
Asymptomatic	101 (14.0%)
Symptomatic, but completely ambulatory	416 (57.6%)
Symptomatic, <50% in bed during the day	149 (20.6%)
Symptomatic, >50% in bed during the day	53 (7.3%)
Bedbound	3 (0.4%)
Had at least one hospitalization in the prior month	138 (14.8%)
Had at least one ER visit in the prior month	60 (6.4%)

^a^ Dual-enrolled into Medicaid/Medicare (n=74, 7.9%).

^b^ CTP class: A (n=141, 17.2%); B (n=475, 57.8%); C (n=206, 25.1%).

^c^ Hepatocellular cancer stage: A (n=60/252, 23.8%); B (n=100/252, 39.7%); C (n=92/252, 36.5%); multifocal HCC with any CTP (n=144/244, 59.0%).

Abbreviations: BMI, body mass index; CCI, Charlson Comorbidity Index; CTP, Child–Turcotte–Pugh; ECOG, Eastern Cooperative Oncology Group; ER, emergency room; HBV, hepatitis B virus; HCC, hepatocellular carcinoma; HMO, health maintenance organization; MAFLD, metabolic dysfunction–associated fatty liver disease; MASH, metabolic dysfunction–associated steatohepatitis; MELD, Model for End-Stage Liver Disease; VA, Veterans Affairs.

### Health-related quality of life

**FIGURE 1:**
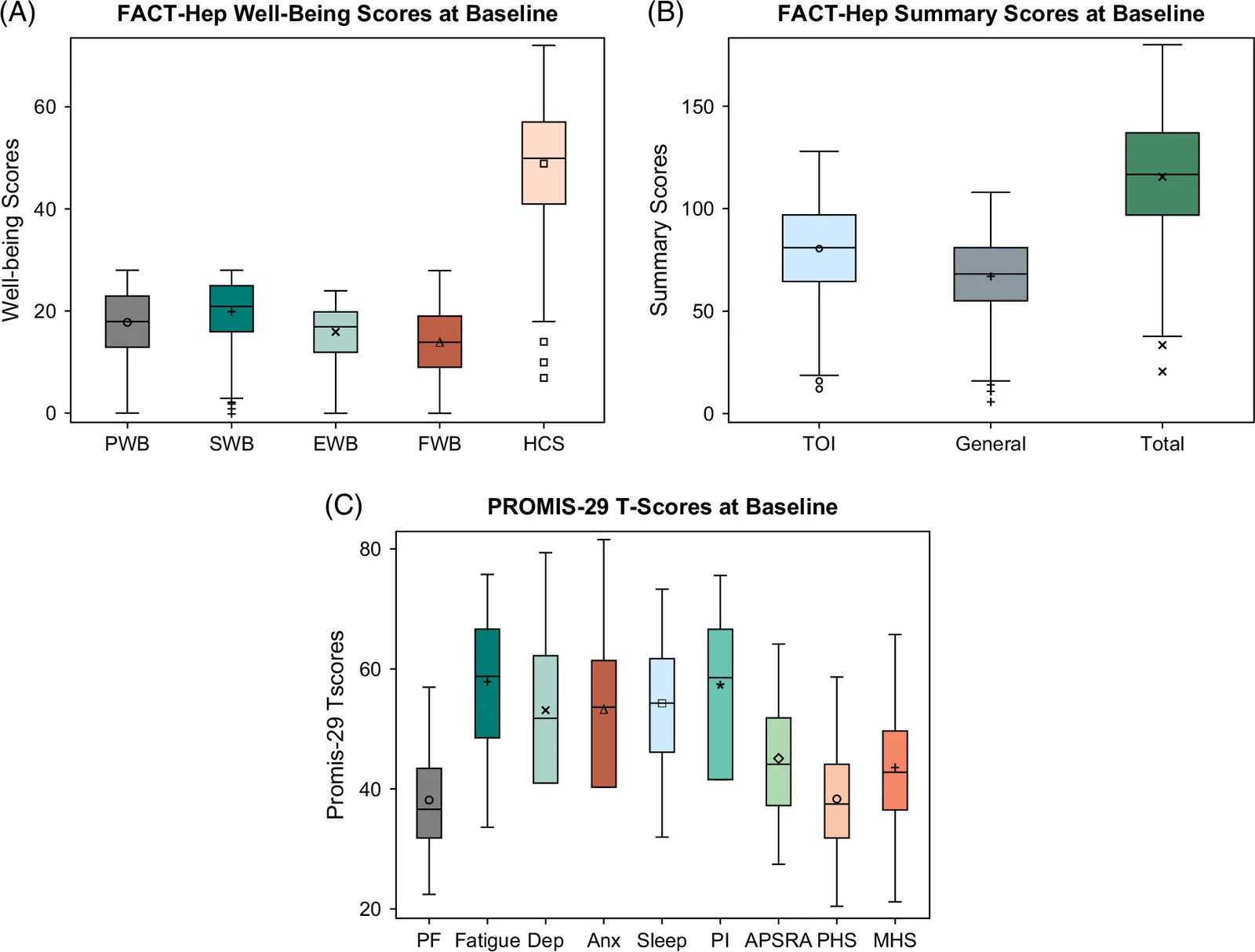
Distribution of FACT-Hep and PROMIS-29 domain and summary scores in the study population. (A) Physical well-being (PWB), social/family well-being (SWB), emotional well-being (EWB), functional well-being (FWB) scores, and hepatobiliary cancer subscale (HCS). (B) FACT-Hep General Score (FACT-G), FACT-Hep Trial Outcome Index (TOI), and FACT-Hep Total Score. (C) PROMIS-29 domain and summary scores: PF (physical function), Dep (depression), Anx (anxiety), PI (pain interference), APSRA (ability to participate in social roles and activities), PHS (physical health score), and MHS (mental health score). The solid line in the box is the median, and the marker in each box is the mean. Outliers appear outside the box and are 1.5 × IQR. Abbreviations: APSRA, ability to participate in social roles and activities; Anx, anxiety; Dep, depression; FACT-G, Functional Assessment of Cancer Therapy–General; FACT-Hep, Functional Assessment of Cancer Therapy–Hepatobiliary; FWB, functional well-being; IQR, interquartile range; MHS, mental health score; PF, physical function; PHS, physical health score; PI, pain interference; PWB, physical well-being; SWB, social/family well-being; TOI, Trial Outcome Index.

The mean (SD, possible range) for FACT-Hep total score was 116 (28.16, 0–180). Subscale and other FACT-hep measurements were as follows: physical well-being (PWB): 18 (6.63, 0–28), social/family well-being (SWB): 20 (6.53, 0–28), emotional well-being (EWB): 16 (5.47, 0–24), functional well-being (FWB): 14 (6.60, 0–28), hepatobiliary cancer subscale (HCS): 49 (11.64, 0–72), FACT-G total score: 67 (18.81, 0–108), and Trial Outcome Index (TOI): 81 (21.79, 0–128) (Figures 1A, B).

Mean (SD) PROMIS-29 domain scores (range 0–100) were as follows: physical health 38 (9.55), anxiety 53 (11.09), depression 53 (10.82), fatigue 58 (11.52), sleep disturbance 54 (10.92), ability to participate in social roles 45 (10.61), and pain interference 57 (11.01). Mean pain intensity was 4 (3.09) (Figure 1C). The mean (SD) PROMIS physical health summary score (PHS) was 38 (9.68), and the mental health summary score (MHS) was 43 (9.59). Both these scores are significantly lower than the general population (mean 50, SD 10) (*p*=0.0001). The mean PROPr score was 0.26 (SD 0.22, 0–1.00).

**FIGURE 2:**
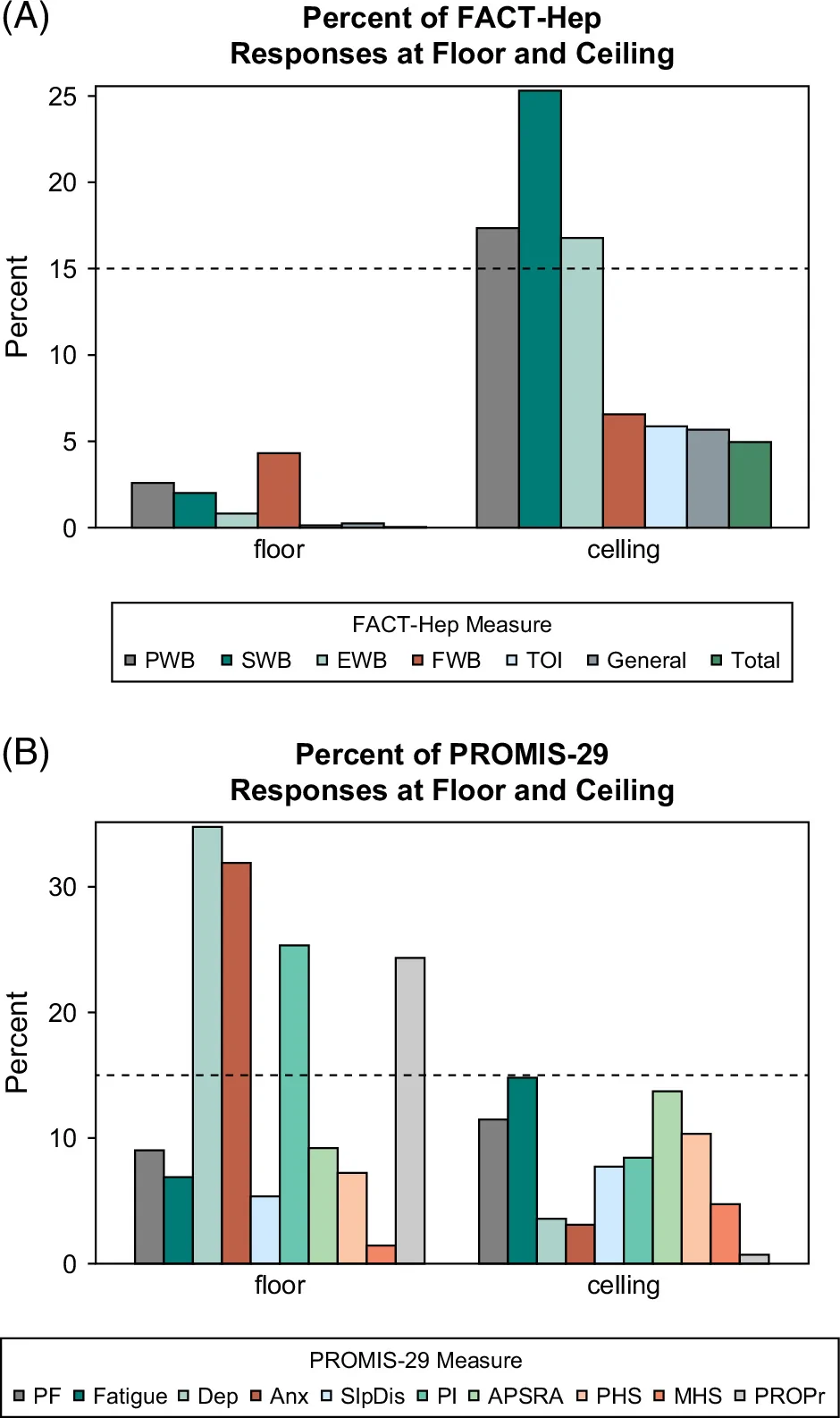
FACT-Hep and PROMIS-29 profile domain and summary score floor and ceiling effects. (A) FACT-Hep and (B) PROMIS-29. When interpreting the PROMIS-29 floor/ceiling effects, it is important to note that smaller scores in fatigue, depression, anxiety, sleep disturbance, pain behavior, and pain intensity domains are associated with better outcomes, and so, floor effects should be interpreted as ceiling effects (limitation in measuring better HRQoL in these domains) while ceiling effects in these domains should be interpreted as floor effects. Abbreviations: APSRA, ability to participate in social roles and activities; Anx, anxiety; Dep, depression; EWB, emotional well-being; FACT-Hep, Functional Assessment of Cancer Therapy–Hepatobiliary; FWB, functional well-being; HRQoL, health-related quality of life; MHS, mental health score; PF, physical function; PHS, physical health score; PI, pain interference; PROPr, PROMIS-Preference; PWB, physical well-being; SlpDis, sleep disturbance; SWB, social/family well-being; TOI, Trial Outcome Index.

Floor and ceiling effects for FACT-Hep and PROMIS-29 are shown in Figure 2. Significant ceiling effects (>15% of responses at the highest score) were observed for FACT-Hep PWB, SWB, and EWB, whereas no significant floor effects were seen for all FACT-Hep measures. For PROMIS-29, no ceiling effects were noted; however, depression, anxiety, and pain interference demonstrated significant floor effects, indicating that a substantial proportion of participants reported the lowest possible burden in these areas, reflecting better HRQoL in these domains.

Significant correlations were observed between FACT-Hep and PROMIS-29 measures (Supplemental Table S2, https://links.lww.com/HC9/C479). The PROPr demonstrated a strong positive correlation with the FACT-Hep total score (Spearman ρ=0.78). PROMIS summary scores were moderately to strongly correlated with FACT-Hep total score (PHS ρ=0.60; MHS ρ=0.82). Domain-level PROMIS T-scores correlated with corresponding FACT-Hep subscales. FACT-Hep SWB showed weak correlations with all PROMIS domains, including the ability to participate in social roles and activities.

### HRQoL in key subgroups

**FIGURE 3:**
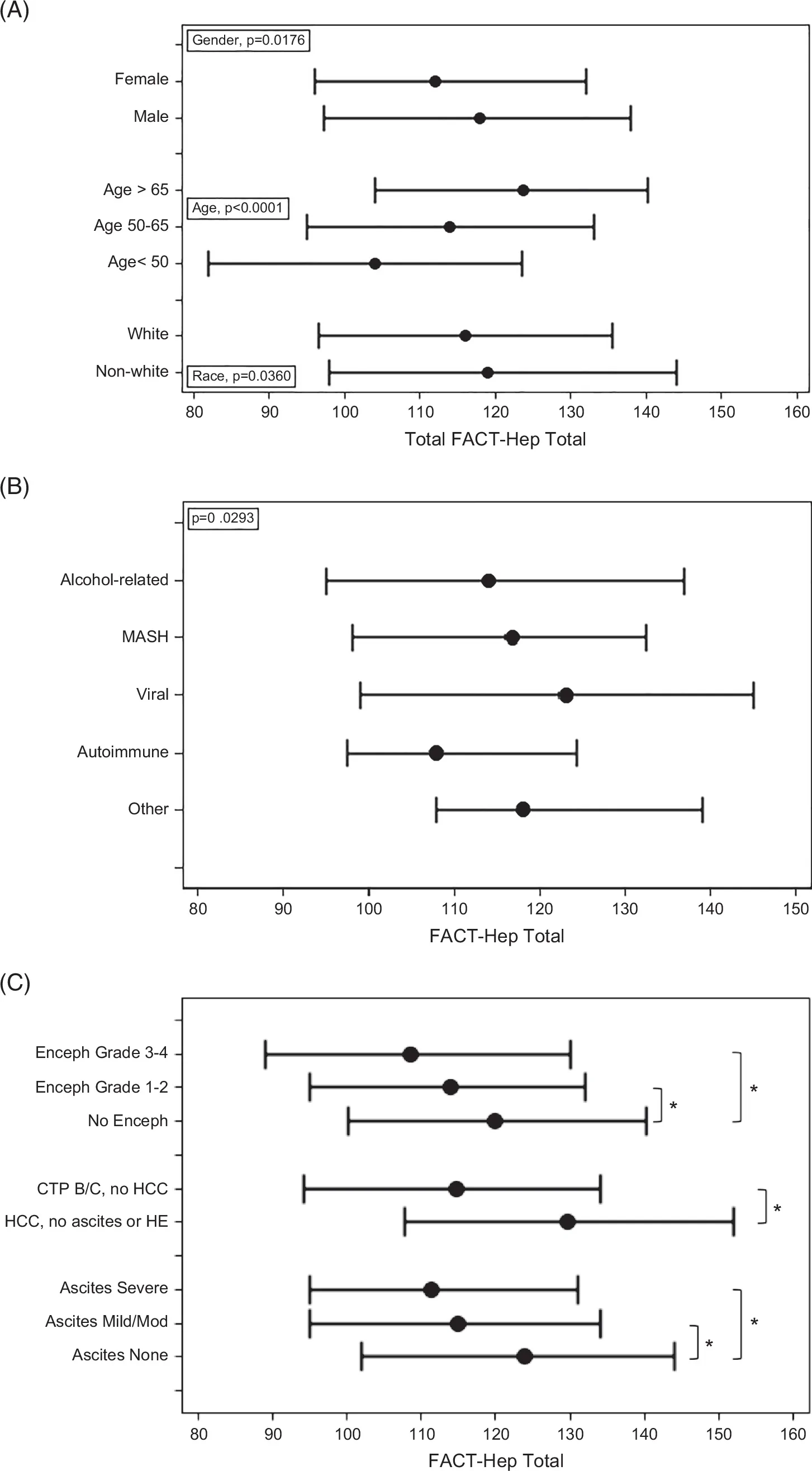
FACT-Hep total score by key sociodemographic and clinical features. (A) By demographics features; (B) By primary liver disease etiology; (C) By cirrhosis complications (* indicates *p*<0.05 for pairwise comparison). Abbreviations: CTP, Child–Turcotte–Pugh; FACT-Hep, Functional Assessment of Cancer Therapy–Hepatobiliary; HCC, hepatocellular carcinoma; HE, hepatic encephalopathy; MASH, metabolic dysfunction–associated steatohepatitis.

Median FACT-Hep total scores were significantly lower among females compared with males, participants younger than 50 compared with >50, and those reporting White versus non-White race (*p*<0.05 for all) (Figure 3). Median FACT-Hep total scores also varied by disease etiology, with the lowest observed among patients with autoimmune cirrhosis, followed by ALD, MAFLD, and the highest among those with viral hepatitis. Notably, patients with ALD had significantly worse EWB and SWB scores compared with those with viral hepatitis.

Patients with ascites or HE had significantly worse FACT-Hep total scores compared with those without these complications (*p*<0.05). Patients with HCC without ascites or HE reported higher HRQoL than those with CTP B/C cirrhosis without HCC (*p*<0.0001). There was no significant difference in scores between mild versus moderate/severe ascites (*p*=0.69); however, scores were lower in those with a history of grades 3–4 HE versus grades 1–2 (*p*=0.0002) (Figure 3). The distribution of other FACT-Hep summary scores (TOI and FACT-G) followed a similar pattern (Supplemental Table S1, https://links.lww.com/HC9/C479, and Supplemental Figure S1, https://links.lww.com/HC9/C478).

In an analogous subgroup analysis, PROMIS-29 summary scores were lower in females (*p*<0.05 for PROPr, PHS, and MHS), those aged <50 (*p*<0.05 for PROPr and MHS), but similar between races (*p*>0.05 except MHS) (Supplemental Figure S2, https://links.lww.com/HC9/C478). These scores were similar when compared by etiologies (*p*>0.05 for PROPr, PHS, and MHS). Consistent with FACT-Hep findings, PROMIS-29 scores were lower in those with ascites and HE, and higher among those with HCC.

Predictors of HRQoL (Supplemental 1: Model Summary, https://links.lww.com/HC9/C477).

Provider-reported ECOG performance status emerged as the strongest predictor of HRQoL (Table 2). Increasing age was associated with higher HRQoL scores; each 10-year increase in age corresponded to nearly a 7-point higher FACT-Hep total score, a 3-point higher PROMIS MHS score, and a 0.04-point higher PROPr score but was not independently associated with the PROMIS PHS score. Female sex was independently associated only with the PROMIS PHS score, with a nearly 2-point lower predicted score compared with males. Smoking was associated with a lower FACT-Hep total score.

**TABLE 2:** Independent predictors of FACT-Hep total score and PROMIS-29 summary scores with ECOG status included

	FACT-Hep total score	PROMIS-PHS	PROMIS-MHS	PROPr^a^
Gender (female)		**−1.86 (−3.66, −0.07)**		
Age (per 10 y)	**7.28 (4.63, 9.92)**		**3.04 (2.15, 3.94)**	**0.42 (0.22, 0.63)**
MELD 3.0 (per 5 points)	**−2.88 (−5.10, −0.65)**			
Marital status (3 df, ref=married)				
Divorced		0.42 (−1.45, 2.29)	0.44 (−1.47, 2.35)	0.10 (−0.34, 0.54)
Single		**2.75 (0.86, 4.64)**	**2.88 (0.94, 4.81)**	**0.75 (0.31, 1.19)**
Widowed		−0.16 (−2.89, 2.58)	0.08 (−2.71, 2.88)	0.02 (−0.62, 0.67)
Ascites (2 df, ref=none)				
Mild/mod		**−2.43 (−4.22, −0.63)**		
Severe		**−3.94 (−6.34, −1.53)**		
CCI*			**−0.38 (−0.74, −0.02)**	
Smoking	**−6.10 (−11.16, −1.04)**			
ECOG status (3 df, ref=asymptomatic)^b^				
>50% in bed	**−22.70 (−32.73, −12.68)**	**−14.67 (−18.00, −11.34)**	**−8.37 (−11.77, −4.97)**	**−2.40 (−3.18, −1.62)**
<50% in bed	**−18.73 (−26.48, −10.97)**	**−9.42 (−11.98, −6.86)**	**−6.13 (−8.74, −3.51)**	**−1.63 (−2.24, −1.03)**
Sx, but ambulatory	**−7.86 (−14.32, −1.39)**	**−3.42 (−5.53, −1.31)**	**−1.70 (−3.86, 0.45)**	**−0.58 (−1.07, −0.08)**

*Note:* Each score (column) was assessed independently. Blank cells indicate that the variables did not achieve statistical significance (α=0.05) in the model for the summary score in question.

Bolded scores indicate *p*<0.05 in the multivariable logistic regression model. Covariates that were included but were not significant in any of these models: race, insurance status, education, encephalopathy, cancer, and primary liver diagnosis. Results are based on the 0.05 level of significance from the F-statistic in type III testing in linear regression models, indicating significance after adjustment for other covariates.

*Calculated without including age and liver disease.

^a^ Change in response to PROPr indicates a change of 0.1 rather than 1 unit for parameter estimates.

^b^ Only 3 participants were classified as bedbound at baseline; therefore, this subgroup was excluded from the analysis.

Abbreviations: CCI, Charlson Comorbidity Index; ECOG, Eastern Cooperative Oncology Group; FACT-Hep, Functional Assessment of Cancer Therapy–Hepatobiliary; MELD, Model for End-Stage Liver Disease; MHS, Mental Health Score; PROMIS, Patient-Reported Outcomes Measurement Information System; PROMIS-MHS, PROMIS–Mental Health Summary; PROMIS-PHS, PROMIS–Physical Health Summary; PROPr, PROMIS-Preference; Sx, symptoms.

MELD showed a stronger relationship with FACT-Hep than with PROMIS measures, particularly when ECOG performance status was included in the model. MELD was significantly associated with FACT-Hep both with ECOG (*p*=0.0114) and without ECOG (*p*=0.0001), while significance for the PROMIS measures (PHS, MHS, and PROPr) was observed only when ECOG was excluded from the model (*p*=0.0033, 0.0059, and 0.0066, respectively). For every 5-point decrease in MELD 3.0 score, the FACT-Hep total score increased by ~3 points.

In models excluding ECOG performance status (Supplemental Table S3, https://links.lww.com/HC9/C479), both PHS and MHS decreased by ~1 point for every 5-point increase in MELD 3.0 score.

Ascites was independently associated with lower PHS, MHS, and PROPr scores (Supplemental Table S3, https://links.lww.com/HC9/C479). BMI was associated with the PROMIS summary scores but not the FACT-Hep score, while the CCI was independently associated only with the MHS score.

## DISCUSSION

In this large, multicenter cohort of patients with ACLD, predominantly characterized by decompensated cirrhosis, HRQoL was substantially impaired, underscoring the pervasive physical and psychosocial burden experienced by this population. This is the first study showing FACT-Hep scores varied by liver disease etiology, functional status, and clinical characteristics, supporting its construct validity and ability to capture disease-related symptom burden and functional impairment central to the lived experience of patients with ACLD. FACT-Hep showed no floor effects, suggesting sensitivity in detecting variation among patients with poor functioning. This indicates that FACT-Hep can be preferred for use in evaluating palliative care interventions, where capturing specific change among lower-functioning patients is critical.

This data extends our knowledge of cirrhosis severity in 5 major ways.

First, FACT-Hep scores differed by liver disease etiology. Particularly, worse emotional and social well-being in ALD suggests the heightened psychosocial impact beyond physical health alone. This is consistent with prior literature demonstrating higher psychological distress, stigma, and social vulnerability among individuals with ALD.^27^,^28^

Second, functional status as measured by ECOG status consistently emerged as a strong predictor of both liver-specific (FACT-Hep) and generic (PROMIS-29) HRQoL.^29^ Worse ECOG status was associated with significantly lower physical and mental health scores, highlighting the profound impact of physical disability on perceived well-being.

Third, MELD 3.0 demonstrated a stronger and more consistent association with FACT-Hep than with PROMIS measures, likely reflecting the disease-specific nature of FACT-Hep in capturing liver-related symptom burden. In contrast, the attenuation of associations with PROMIS measures after adjustment for ECOG performance status suggests that broader health domains measured by PROMIS may be more strongly influenced by overall functional status than by liver disease severity alone.

Fourth, smoking was independently associated with worse HRQoL, indicating that modifiable behavioral factors contribute meaningfully to symptom burden beyond liver disease severity. Smoking may act through increased systemic inflammation and oxidative stress, as well as exacerbation of fatigue and frailty, and may also reflect a higher burden of comorbidities and adverse psychosocial factors independently linked with reduced HRQoL.

Finally, very few clinical or demographic variables were significantly associated with HRQoL scores. This underscores that HRQoL captures a distinct and largely independent dimension of patient experience that is not reflected in routinely collected clinical measures and is therefore not currently represented in standard clinical care for ACLD populations.

The higher FACT-Hep scores observed among patients with viral hepatitis relative to those with autoimmune liver disease should be interpreted cautiously. Potential explanations include differences in symptom burden, treatment requirements, extrahepatic manifestations, and perceptions of disease control; however, these factors were not specifically evaluated in this study. Future studies examining disease-specific determinants of health-related quality of life may help clarify these findings.

### Convergent validity of FACT-Hep and PROMIS-29

The strong correlation between PROMIS-29 summary scores and FACT-Hep total score (ρ ranging from 0.7 to 0.8) supports the construct validity. Domain-level analyses further demonstrated conceptual alignment. FACT-Hep EWB correlated strongly with PROMIS depression and anxiety, while physical and functional domains showed moderate correlations with PROMIS fatigue, pain interference, and sleep disturbance. These findings indicate substantial overlap in the measurement of core physical and psychological constructs across instruments.

In contrast, the FACT-Hep SWB demonstrated weak correlations with PROMIS domains, including the ability to participate in social roles and activities, likely reflecting differences in conceptual focus. FACT-Hep SWB emphasizes perceived social support and illness acceptance (eg, “I get support from my friends” or “My family has accepted my illness”), whereas PROMIS social function primarily assess role functioning and participation in usual activities (eg, “I have trouble doing all of my usual work” or “I have trouble doing all of the activities with friends that I want to do”). Thus, FACT-Hep may capture liver disease–specific social and interpersonal stressors that are not fully reflected in PROMIS constructs.

From a practical perspective, the 45-item FACT-Hep imposes greater respondent burden than shorter instruments such as PROMIS-29 or the CLDQ. Consequently, PROMIS-29 may be preferable when a brief, generic assessment of HRQoL is desired. In contrast, FACT-Hep may be particularly valuable in studies or clinical settings where detailed assessment of liver disease–specific symptoms and concerns is important.

### Effect modification by age

Increasing age was independently associated with higher HRQoL across both FACT-Hep and PROMIS-29 measures. Although seemingly counterintuitive, this finding may reflect a form of psychological adaptation, response shift, or recalibration of health expectations among older patients.^30^

The persistence of this association after multivariable adjustment underscores that perceived HRQoL is shaped not only by disease severity but also by psychosocial context and life stage.

In addition, the relative contribution of physical and emotional domains to overall HRQoL may vary by age, suggesting differences in symptom appraisal, prioritization, and response shift. Age-related differences in symptom appraisal and prioritization should be considered when interpreting PROMs and comparing HRQoL across cirrhosis populations.^30^

### Clinical and research implications

These findings have important implications for integrating PROMs into the care and study of patients with ACLD, particularly decompensated cirrhosis. Both FACT-Hep and PROMIS-29 demonstrated validity and clinical relevance, with overlapping yet distinct contributions. Instrument selection should therefore be guided by the study or clinical objective.

FACT-Hep provides disease-specific insight into hepatic symptoms, functional impairment, and social stressors, making it particularly well-suited for studies or interventions targeting decompensated cirrhosis or HCC. With only ceiling effects in PWB, SWB, and EWB, indicating limitations in measuring the highest HRQoL, this instrument may be the key resource needed to identify unmet needs of those with poor HRQoL in clinical practice.^31^ In fact, the lack of any floor or ceiling effects in the summary scores of FACT-Hep (total, general, TOI) means that this tool can measure HRQoL across a wide spectrum of symptom burden, allowing detection of meaningful changes in HRQoL. PROMIS-29, in contrast, captures broader physical, mental, and social health domains, facilitating comparisons across disease states and benchmarking against general population norms. While we noted some floor effects in the depression, anxiety, and pain interference PROMIS domains, these indicate some limitations in measuring those with minimal symptoms in these domains. Although there were no floor or ceiling effects in the PROMIS-29 summary scores, as with FACT-Hep summary scores, the floor effects in our derived PROPr scores need further attention before use in populations with high symptom burden, such as those included in this study. Taken together, these instruments offer complementary perspectives: FACT-Hep ensures sensitivity to liver-specific impacts, while PROMIS-29 allows HRQoL measurement to be placed within the context of both the general population and other chronic diseases.

In clinical practice, selective use of key domains from either instrument may balance comprehensive assessment with minimization of responder burden. The strong association between ECOG performance status and HRQoL underscores the utility of functional status as a practical proxy in care planning and prognostication. The association between MELD 3.0 and PROMIS measures was attenuated after adjustment for ECOG performance status, suggesting that functional status may be more closely associated with patient-reported outcomes than laboratory-based measures of disease severity. Etiology-specific differences further highlight the need for tailored supportive care strategies, particularly for patients with ALD. Our findings should spur investigation into the key overlooked symptoms driving these deficits, including management of chronic pain and sexual function.^32^,^33^ Finally, the independent associations of smoking, comorbidity burden, and BMI with HRQoL highlight potentially modifiable contributors beyond hepatic dysfunction.

Systematic integration of PROMs into hepatology practice may facilitate identification of patients with high symptom burden, guide supportive care interventions, including ascites management and behavioral risk modification, and advance patient-centered care beyond traditional laboratory-based metrics.

### Limitations

These data must be interpreted in the context of the study design. First, the cross-sectional design precludes assessment of longitudinal responsiveness and limits inference regarding the temporal relationship between liver disease progression and changes in HRQoL. As such, we cannot determine causality or evaluate the sensitivity of these instruments to clinical change over time. Although missing PROM data were not imputed, and missingness may have been higher among patients with worse functional status, the relatively low proportion of missing data is unlikely to have meaningfully affected the overall findings.

Second, although ECOG performance status emerged as the strongest predictor of HRQoL, it is a provider-reported measure and may conceptually overlap with patient-reported physical function domains. This overlap may partially attenuate observed associations between liver-specific severity markers (eg, MELD 3.0) and HRQoL in multivariable models.

Third, although this study included a large, multicenter cohort, the population was predominantly composed of patients with decompensated cirrhosis with projected life expectancy >3 months and enrolled in a clinical research setting. These characteristics may limit generalizability to patients with compensated disease, severe phenotypes, or those receiving care outside tertiary centers. Fourth, ascites severity was classified using baseline clinical data and did not capture more detailed measures of disease burden, such as the need for serial large-volume paracentesis, refractory ascites, or longitudinal changes in ascites status. As these factors may substantially influence patient-reported quality of life, future studies incorporating more granular assessments of ascites severity may provide additional insights into the relationship between portal hypertension complications and HRQoL.

Fifth, missing data were not imputed, which may limit generalizability. However, the proportion of missing HRQoL data at baseline was modest (<12%), mitigating the potential impact. Finally, unmeasured factors, including socioeconomic stressors, prior mental health diagnoses, and severity of substance use, may also influence HRQoL but were not fully captured in this analysis. The Chronic Liver Disease Questionnaire (CLDQ) was not utilized in this trial, and thus, its performance could not be compared with FACT-Hep. In addition, we did not capture the time it took participants to complete any of the HRQoL measures, although, on average, as reported by study coordinators, it did not take more than twenty minutes to complete HRQoL instruments.

## CONCLUSIONS

In summary, this study demonstrates that both FACT-Hep and PROMIS-29 are valuable tools for assessing HRQoL in patients with decompensated cirrhosis, with meaningful correlations between instruments and significant associations with clinical and demographic factors. FACT-Hep should be used for detailed characterization of disease burden as an ideal symptom-based HRQoL instrument, while PROMIS-29 offers breadth and broad comparability.

Future longitudinal studies are needed to evaluate how HRQoL evolves over time, particularly in relation to hepatic decompensation events, disease progression, and supportive care interventions, and to determine whether PROM-guided approaches can improve clinical and patient-centered outcomes in advanced liver diseases. Future studies should also compare FACT-Hep with other established liver disease–specific instruments.

## Supplementary Material
