# Supplementary material for: Measuring quality of life in advanced chronic liver disease with FACT-Hep: Findings from the PAL LIVER trial
Source: Hepatol Commun. 2026 Aug 28;10(9):e1043. doi: 10.1097/HC9.0000000000001043 (PMC13533500; doi:10.1097/HC9.0000000000001043)

Supplemental Figure 1. FACT-Hep Trial Outcome Index and General Score by key sociodemographic and clinical features. Panel A: By demographics features; Panel B: By Primary Liver Disease Etiology; Panel C: By Cirrhosis Complications. (* indicates p <0.05 for pairwise comparison)

Supplement: Figure 1 – Panel A: FACT-Hep TOI and General across demographics

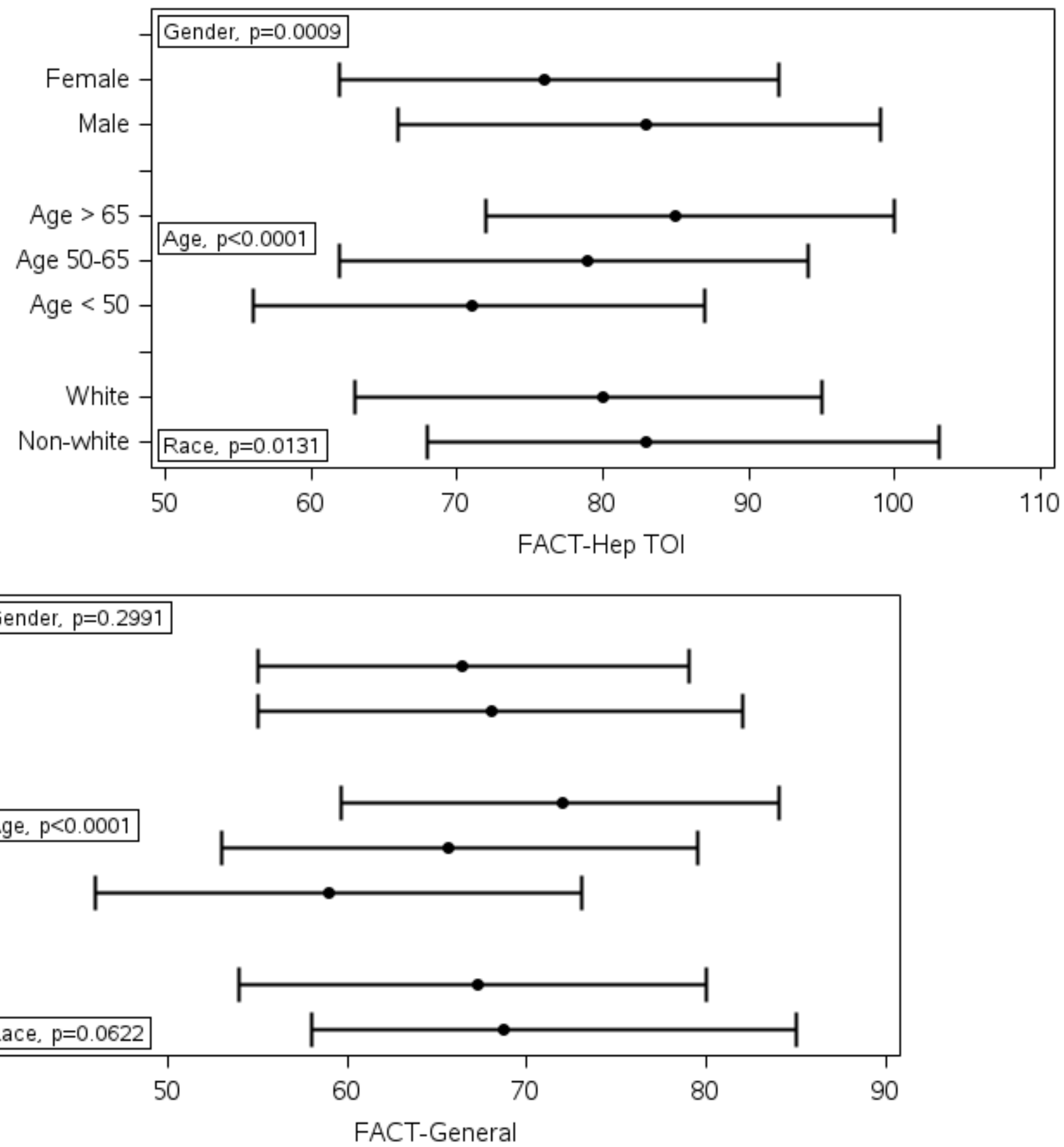

Supplement: Figure 1 – Panel B: FACT-Hep TOI and General across Liver Disease Etiology

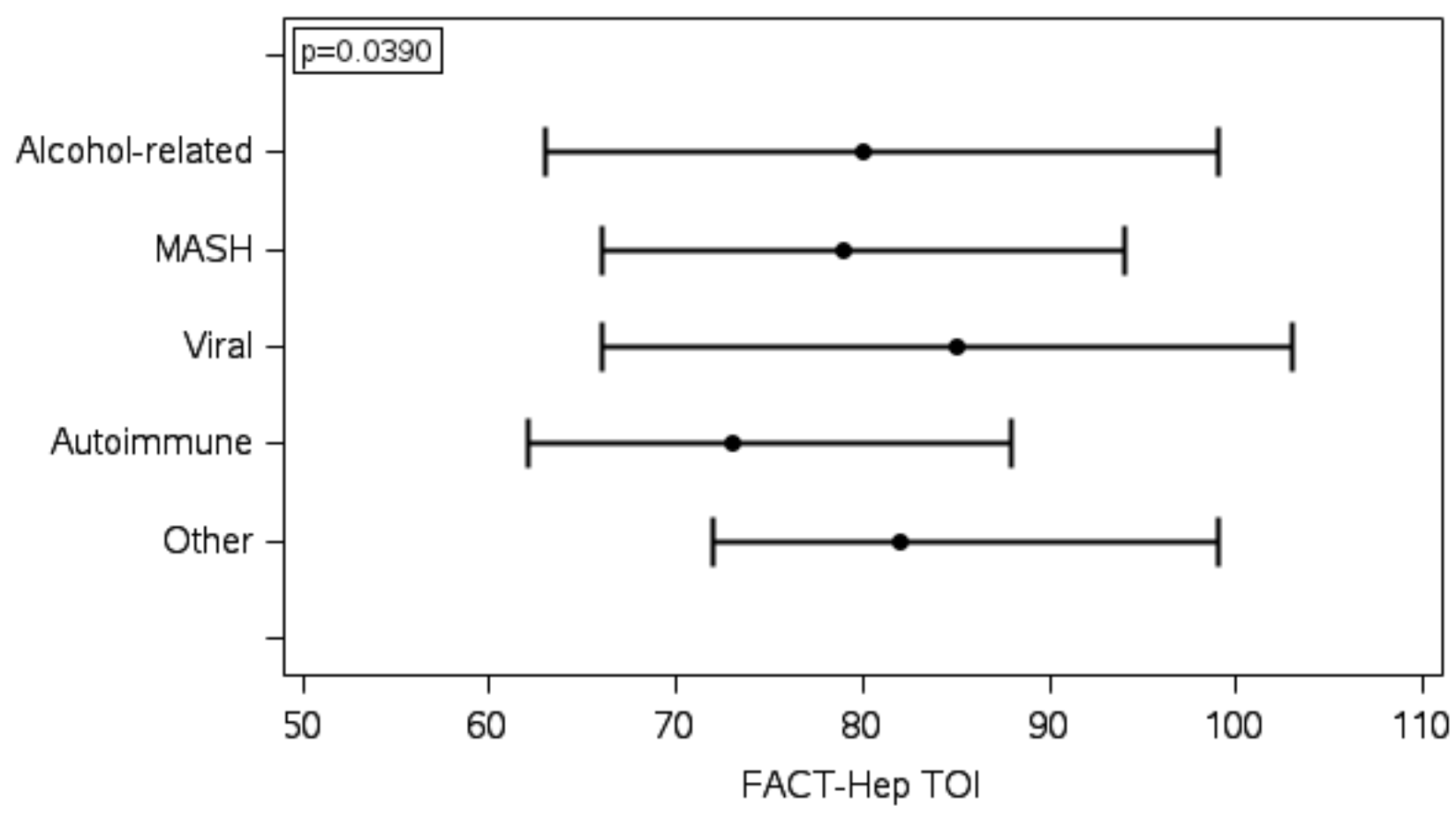
p=0.0390
Alcohol-related
MASH
Viral
Autoimmune
Other
50
60
70
80
90
100
110
FACT-Hep TOI

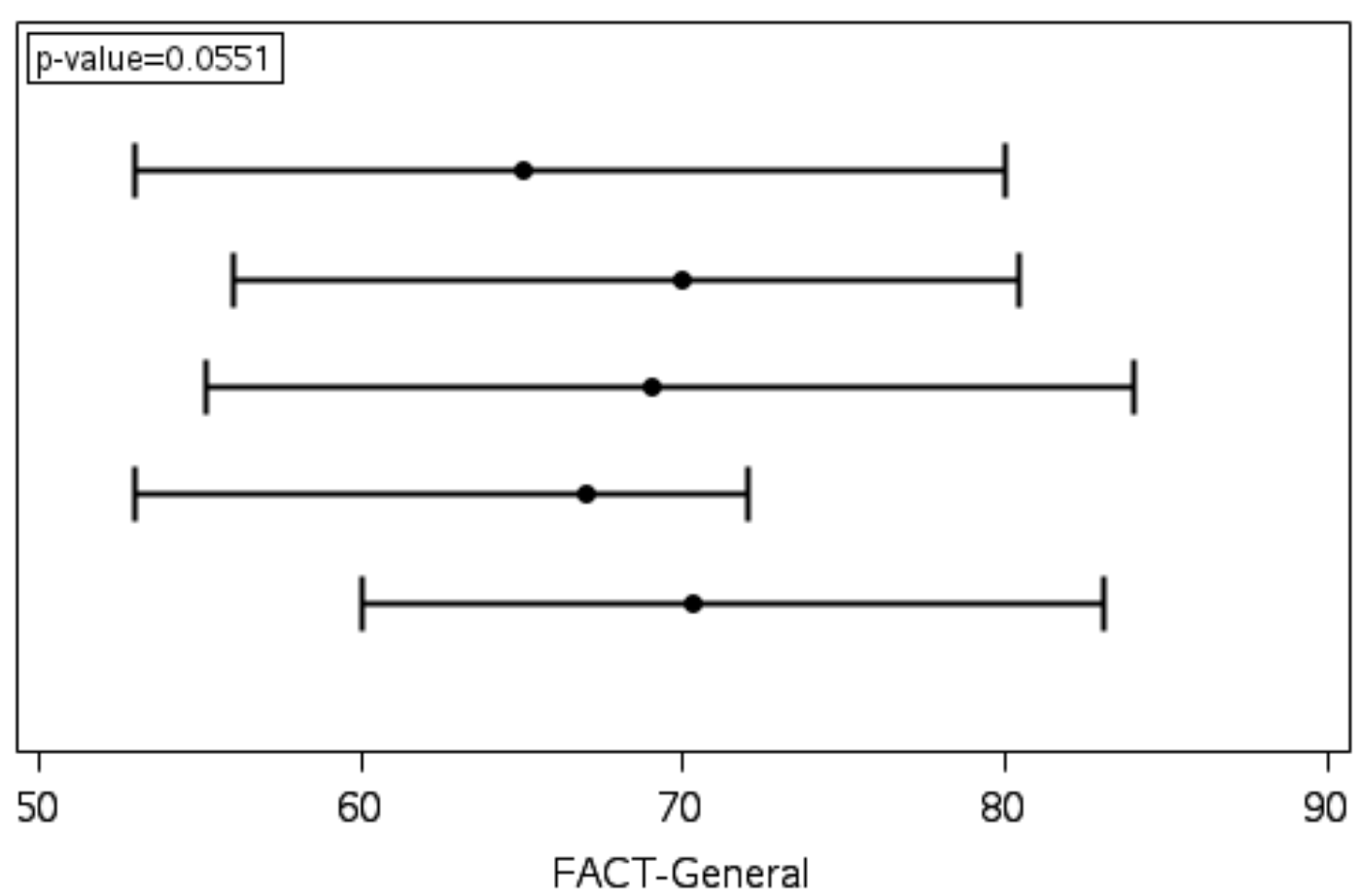
p-value=0.0551
50
60
70
80
90
FACT-General

Supplement: Figure 1 – Panel C FACT-Hep TOI and General across Liver Disease Complications

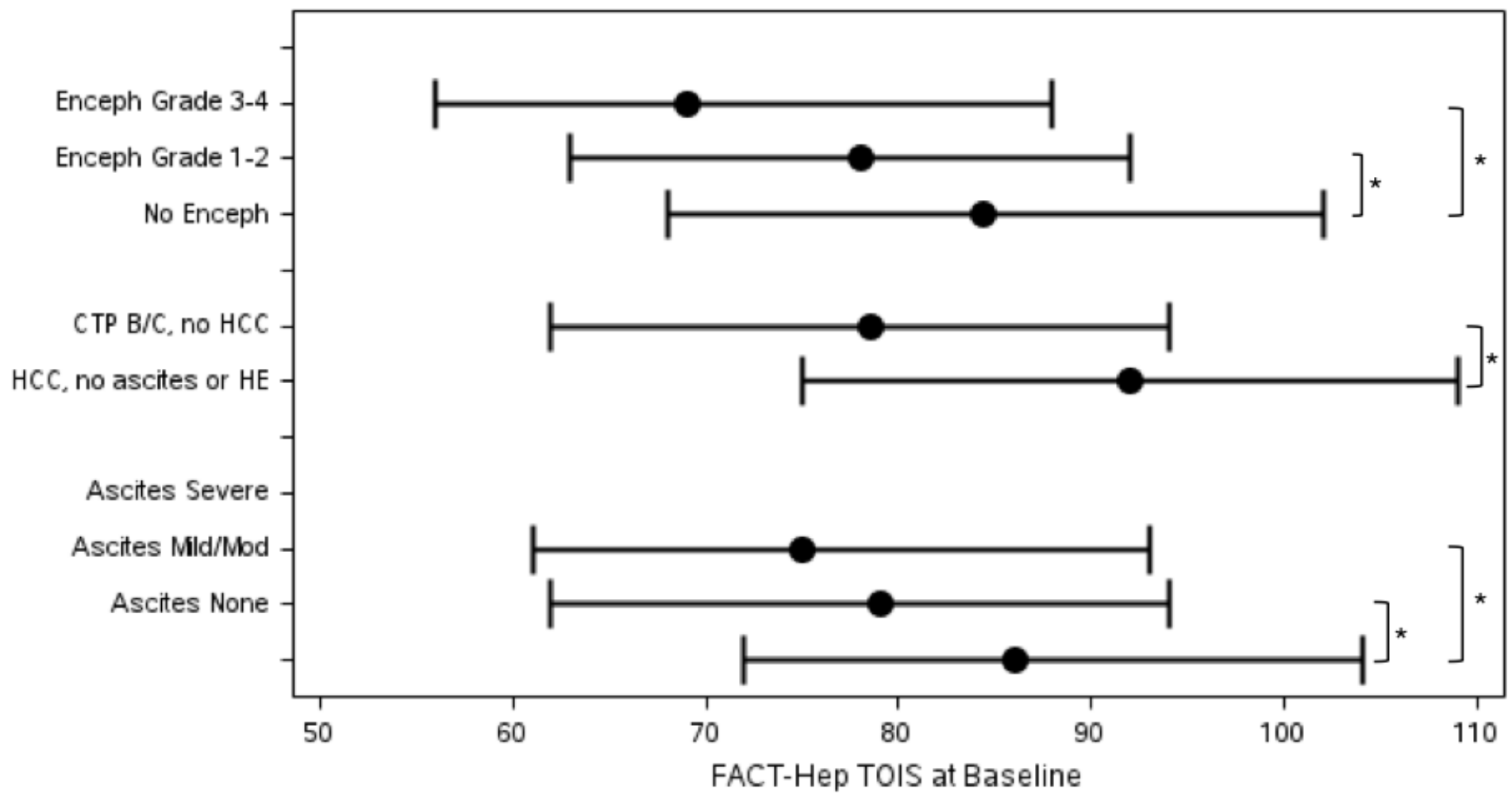

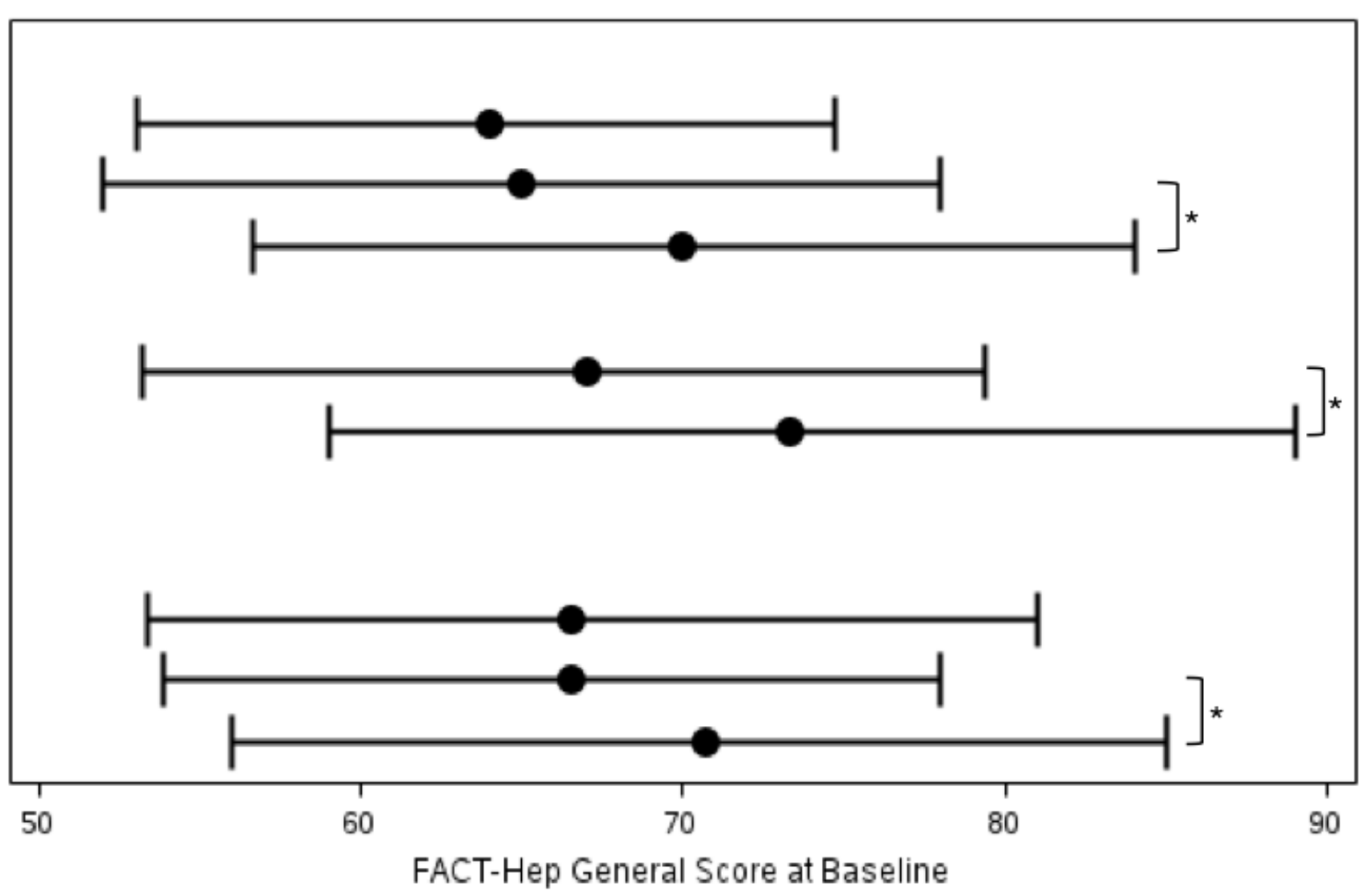

Supplementary Figure 2. PROMIS-29 Profile PROPr, PCS and MCS Scores by key sociodemographic and clinical features. Panel A: By demographics features; Panel B: By Primary Liver Disease Etiology; Panel C: By Cirrhosis Complications. (* indicates p <0.05 for pairwise comparison)

Supplement: Figure 2 – Panel A: PROMIS-29 PROPr, Physical Health and Mental Health across Demographics (3 plots)

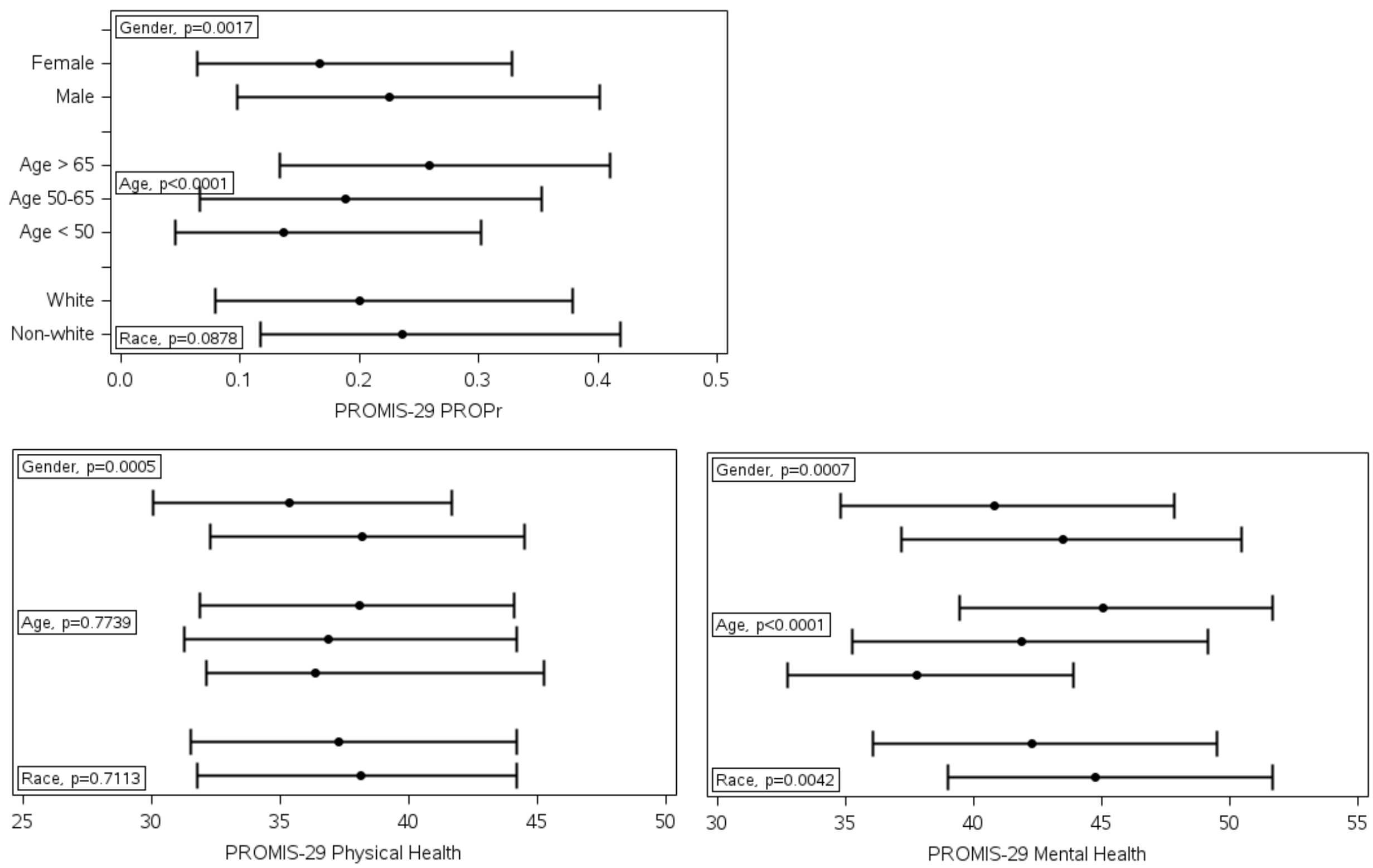

Supplement: Figure 2 – Panel B: PROMIS-29 PROPr, Physical Health and Mental Health across Liver Disease Etiology (3 plots)

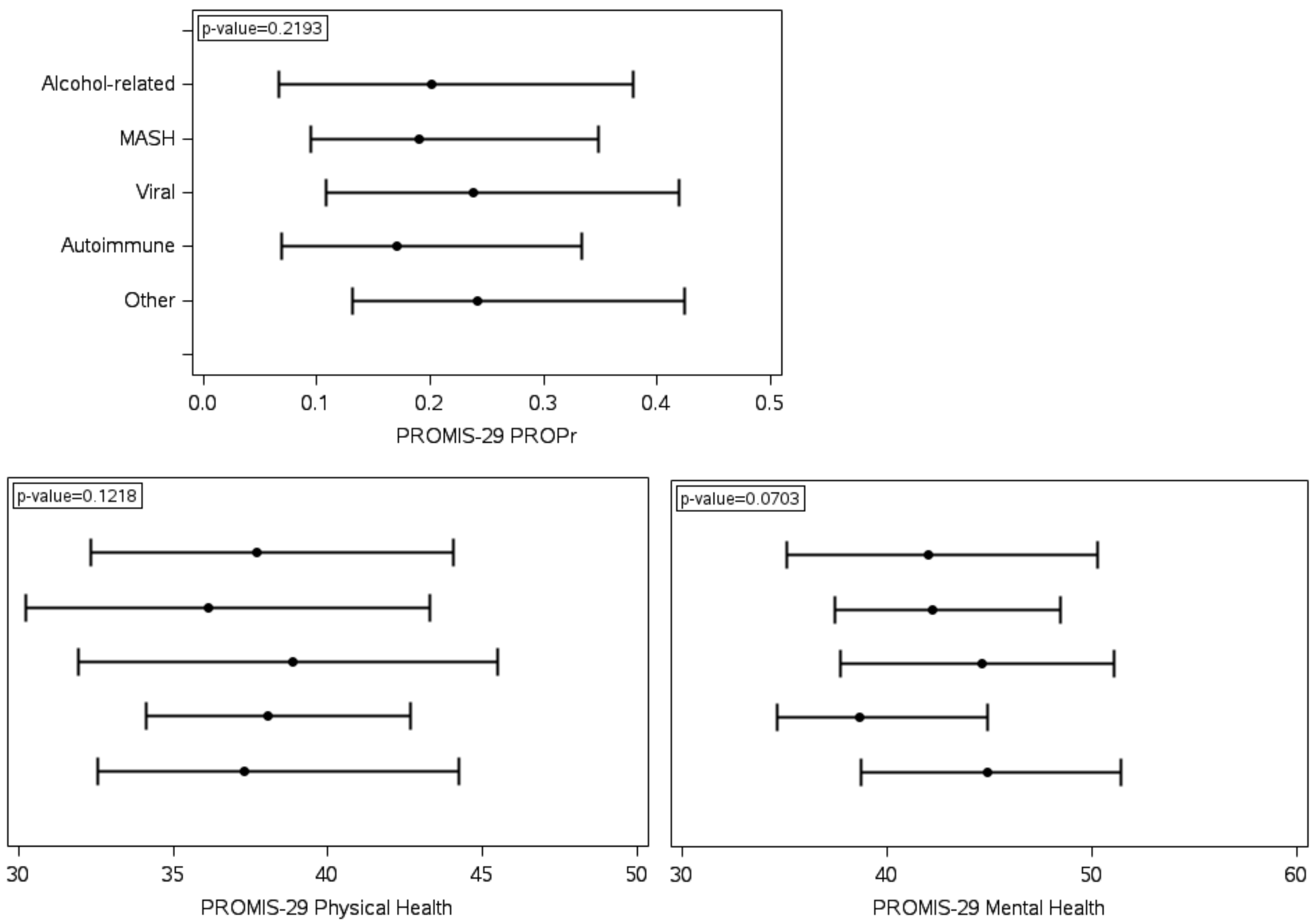

Supplement: Figure 2 – Panel C: PROMIS-29 PROPr, Physical Health and Mental Health across HCC/Cirrhosis Complications

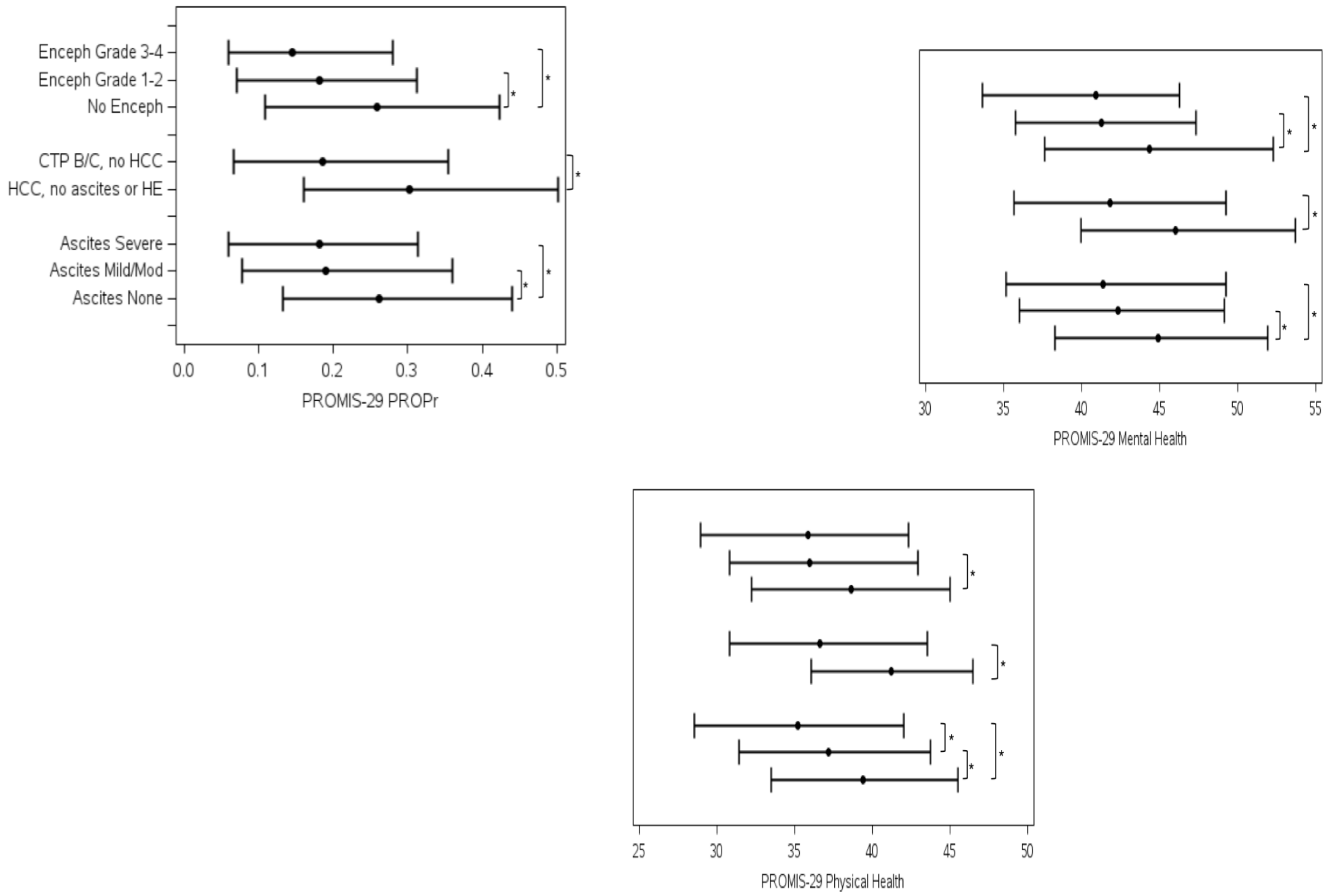

Supplement: Supplementary file 1 [file hc9-10-e1043-s002.pdf]
