# Supplementary material for: Measuring quality of life in advanced chronic liver disease with FACT-Hep: Findings from the PAL LIVER trial
Source: Hepatol Commun. 2026 Aug 28;10(9):e1043. doi: 10.1097/HC9.0000000000001043 (PMC13533500; doi:10.1097/HC9.0000000000001043)
Supplement: Supplementary file 2 [file hc9-10-e1043-s003.pdf]

Supplementary Table 1. Summary of FACT-Hep and PROMIS-29 domain and summary scores by key sub-groups.

	Full Cohort (n=935)	CTP Class B/C without HCC (n= 534)	HCC without Ascites or HE (n= 173)
**FACT-Hep* [Median (IQR)]**			
**Physical Well-being**	18.0 (13.0,23.0)	18.0 (13.0,23.0)	21.0 (16.0,26.0)
**Social Well-being**	21.0 (16.0,25.0)	21.0 (16.0,25.0)	21.0 (15.0,25.0)
**Emotional Well-being**	17.0 (12.0,20.0)	16.0 (12.0,20.0)	18.0 (13.0,21.0)
**Functional Well-being**	14.0 (9.0,19.0)	13.0 (8.0,18.0)	16.0 (11.0,21.0)
**Hepatobiliary Cancer Scale**	50.0 (41.0,57.0)	48.0 (39.0,55.0)	56.0 (45.0,63.0)
**Total Score**	117.0 (97.0,137.0)	114.8 (94.2,134.0)	129.7 (107.8,152.0)
**General Score**	68.0 (55.0,81.0)	67.0 (53.2,79.3)	73.3 (59.0,89.0)
**Trial Outcome Index**	81.0 (64.6,97.0)	78.6 (62.0,94.0)	92.0 (75.0,109.0)
**PROMIS-29***			
**PROPr Score**	0.21 (0.08,0.38)	0.19 (0.07,0.35)	0.30 (0.16,0.50)
**Physical Health Score**	37.5 (31.6,44.2)	36.6 (30.8,43.5)	41.2 (36.1,46.5)
**Mental Health Score**	42.8 (36.6,49.7)	41.8 (35.7,49.2)	46.0 (40.0,53.7)

* Missingness: 9% for FACT-Hep domains/summary scores and 12% for PROMIS summary scores
Values are presented as Median (IQR)

Supplementary Table 2. Correlations between FACT-Hep and PROMIS-29 Domain and Summary Scores. Yellow and green highlighting indicate moderate and strong correlations, respectively.

	FACT-Hep Scores					
**PROMIS Score**	**Total Score**	**PWB**	**EWB**	**SWB**	**FWB**	**HCS**
**Physical function**	0.534	0.553	0.298	0.111	0.536	0.484
**Fatigue**	-0.670	-0.699	-0.471	-0.138	-0.564	-0.634
**Depression**	-0.645	-0.513	-0.664	-0.292	-0.515	-0.507
**Anxiety**	-0.601	-0.461	-0.647	-0.312	-0.451	-0.488
**Sleep Disturbance**	-0.506	-0.387	-0.377	-0.190	-0.501	-0.463
**Pain interference**	-0.611	-0.640	-0.430	-0.178	-0.488	-0.559
**APSRA***	0.610	0.578	0.435	0.130	0.610	0.546
**Pain Intensity**	-0.496	-0.564	-0.329	-0.109	-0.335	-0.486
**Physical Health**	0.603	0.626	0.352	0.130	0.585	0.552
**Mental Health**	0.819	0.770	0.641	0.235	0.699	0.734
**PROPr**	0.780	0.757	0.560	0.208	0.703	0.689

*APSRA: ability to participate in social roles and activities

Color coding: white- *weak < 0.5*, yellow- *moderate 0.5 - 0.7*, green- *strong >= 0.7.*

Supplementary Table 3. Independent Predictors of FACT-Hep Total Score and PROMIS-29 Summary Scores without ECOG status. Blank cells indicate that the variables did not achieve statistical significance (α=.05) in the model for the summary score in question.

	FACT-Hep Total	PROMIS- PHS	PROMIS-MHS	PROPr‡
Gender (female)		**-2.07 (-3.98, -0.15)**		
Age (per 10 yr)	**6.53 (3.93, 9.12)**		**2.81 (1.92,3.70)**	**0.35 (0.14,0.55)**
MELD (per 5 points)	**-4.22 (-6.38, -2.06)**	**-1.15 (-1.91, -0.38)**	**-1.03 (-1.77, -0.30)**	**-.24 (-0.41, -0.07)**
Ascites (2df, ref=none)				
Mild/Mod		**-2.67 (-4.60, -0.74)**		-0.36 (-0.79, 0.07)
Severe		**-5.43 (-8.02, -2.85)**		**-0.82, (-1.40, -0.24)**
BMI		**-0.24 (-0.35, -0.14)**	**-0.11 (-0.22, -0.01)**	**-0.04 (-0.06, -0.02)**
CCI*			**-0.46 (-0.82, -0.09)**	
Smoking	**-7.33 (-12.42, -1.04)**	**-2.43 (-4.23, -0.62)**		

Bolded scores indicate p<0.05 in multivariable logistic regression model.

* calculated without including age and liver disease

‡ Change in response to PROPr indicates change of .1 rather than 1 unit for parameter estimates
