# Supplementary material for: Measuring quality of life in advanced chronic liver disease with FACT-Hep: Findings from the PAL LIVER trial
Source: Hepatol Commun. 2026 Aug 28;10(9):e1043. doi: 10.1097/HC9.0000000000001043 (PMC13533500; doi:10.1097/HC9.0000000000001043)
Supplement: Supplementary file 3 [file hc9-10-e1043-s001.docx]

Model Summary

Linear regression including all covariates

Dependent variables: FACT-Hep, PROMIS Phys Health, PROMIS Mental Health, PROPr

Linear regression modeling was used to assess the association between patient characteristics and four QoL outcomes including FACT-Hep Total, PROMIS-29 Physical Health, PROMIS-29 Mental Health, and PROPr. A higher value of the QoL measure indicates better outcome. No data imputations were performed for missing data.

Fifteen independent covariates selected in either a stepwise or backwards elimination process or thought to have clinical importance were examined in this setting: gender, age, race, marital status, insurance, education, ascites, BMI, MELD, encephalopathy, cancer, Charlson comorbidity index, smoking, primary liver disease diagnosis and ECOG status. Nine of these characteristics were determined to have statistical significance in at least one of these four endpoints (Table 1). Significant and borderline significant relationships between characteristics and outcomes appear in Table 2.

The strongest predictor in all models was ECOG status. ECOG status has levels: asymptomatic, symptomatic but ambulatory, in bed <50% of the time, in bed >50% of the time, bedbound and death. At baseline, no patients were deceased and only 3 were bedbound. These 3 patients were removed from the modeling assessment due to small numbers. Because of the strong impact of ECOG status in the models, the models were also run without ECOG in the candidate list to allow examination of other potential relationships that might be masked due to collinearity. Consistently, as ECOG worsened (increased), the QoL worsened (decreased), regardless of the outcome being assessed. For FACT-Hep total score, with asymptomatic as the reference group, patients in bed >50% of the time experienced a decrease in FACT-Hep total score of 23, while patients in bed <50% were associated with a 19 point decrease in total score and symptomatic patients that were ambulatory were associated with an 8 point decrease in FACT-Hep total score. Similar relationships were observed for the other QoL measures and ECOG.

Age was a significant predictor of outcome in three of the four models, both with and without ECOG in the model. **For FACT-Hep total score, a 10-year increase in age was associated with an increase of 7.3 in total score.** With increase in age, better QoL outcomes were observed. While age was not significant for the PROMIS physical health score outcome, it was significant in predicting mental health score and the PROPr summary variable.

MELD was a significant predictor of outcome for all QoL measures (especially FACT-Hep total score), however for the PROMIS-29 summary measures, it achieved statistical significance only after ECOG was removed from the model. A higher value for MELD indicates worse disease. Consistently, higher MELD values were associated with a decrease in FACT-hep scores.

Marital status was not statistically significant for the dependent variable FACT-Hep Total score, however it was significant in the three PROMIS-29 outcome models that included ECOG status. The parameter estimates (Table 2) use ‘married’ as the reference group. Single vs married is the primary driver of significance in this relationship, with being single associated with slight improvement of QoL.

Smoking, severe ascites, increased Charlson comorbidities and BMI were associated with decreased QoL (Table 2).

Female gender was associated with worse outcomes for only the Physical Health Score outcome (with and without ECOG).

**Table 1**: Variables that achieved statistical significance in at least one of the linear regression models

|  | FACT-Hep Total | | PROMIS Physical Health | | PROMIS Mental Health | | PROPr Summary | |
| --- | --- | --- | --- | --- | --- | --- | --- | --- |
|  | With EC status | Without EC status | With EC status | Without EC status | with EC status | Without EC status | with EC status | Without EC status |
| Gender |  |  | x | x |  |  |  |  |
| Age | x | x |  |  | x | x | x | x |
| MELD | x | x |  | x |  | x |  | x |
| Marital status |  |  | x |  | x |  | x |  |
| Ascites |  |  | x | x |  |  |  | x |
| BMI |  |  |  | x |  | x |  |  |
| CCI (no age or lv dz) |  |  |  |  | x | x |  |  |
| Smoking | x | x |  | x |  |  |  |  |
| ECOG (EC) status | x |  | x |  | x |  | x |  |

Covariates that were included but were not significant in any of these models: race, insurance status, education, encephalopathy, cancer, primary liver diagnosis; Results are based on the .05 level of significance from the F-statistic in Type III testing in linear regression models, indicating significance after adjustment for other covariates.

**Table 2**: Significant Covariates in the Full Model (parameter estimates with 95% CI ). Characteristics without highlights were significant at the alpha-.05 level and those highlighted in blue indicate a borderline significant relationship with outcome.

|  | FACT-Hep Total | | PROMIS Physical Health | | PROMIS Mental Health | | PROPr Summary | |
| --- | --- | --- | --- | --- | --- | --- | --- | --- |
|  | With EC status | Without EC status | With EC status | Without EC status | with EC status | Without EC status | with EC status | Without EC status |
| **Gender (female)** |  |  | -1.86  (-3.66,  -0.07 | -2.07  (-3.98,  -0.15) |  |  |  |  |
| **Age (per 10 yr)** | 7.28  (4.63,9.92) | 6.53  (3.93, 9.12) |  |  | 3.04  (2.15,3.94) | 2.81  (1.92,3.70) | 0.42  (0.22,0.63) | 0.35  (0.14,0.55) |
| **MELD (per 5 unit)** | -2.88  (-5.10,  -0.65) | -4.22  (-6.38,  -2.06) |  | -1.15  (-1.91,  -0.38) |  | -1.03  (-1.77,  -0.30) |  | -.24  (-0.41,  -0.07) |
| **Marital status (ref=married)** |  |  |  |  |  |  |  |  |
| **Divorced** |  |  | 0.42  (-1.45,  2.29) |  | 0.44  (-1.47,  2.35) |  | 0.10  (-0.34,  0.54) | *0.10*  *(-0.35,*  *0.56)* |
| **Single** |  |  | 2.75 (0.86,4.64) |  | 2.88  (0.94,4.81) |  | 0.75  (0.31,1.19) | *0.59*  *(0.13,*  *1.04)* |
| **Widowed** |  |  | -0.16  (-2.89,  2.58) |  | 0.08  (-2.71,  2.88) |  | 0.02  (-0.62,  0.67) | *0.04*  *(-0.63,*  *0.70)* |
| **Ascites (ref=none)** |  |  |  |  |  |  |  |  |
| **Mild/Mod** |  | *-4.86*  *(-10.30,*  *0.57)* | -2.43  (-4.22,  -0.63) | -2.67  (-4.60,  -0.74) |  | *-0.88*  *(-2.74,*  *0.98)* |  | -0.36  (-0.79,  0.07) |
| **Severe** |  | *-8.19*  *(-15.47,*  *-0.92)* | .3.94  (-6.34,  -1.53) | -5.43  (-8.02,  -2.85) |  | *-2.79*  *(-5.28,*  *-0.30)* |  | -0.82,  (-1.40,  -0.24) |
| **BMI** |  | *-0.29*  *(-0.59,*  *0.01)* |  | -0.24  (-0.35,  -0.14) |  | -0.11  (-0.22,  -0.01) | *-0.02*  *(-.05,*  *0.00)* | -0.04  (-.0.06,  -0.02) |
| **CCI (no age or lv dz)** |  | *-0.97*  *(-2.03, 0.10)* |  |  | -0.38  (-0.74,  -0.02) | -0.46  (-0.82,  -0.09) |  | *-0.08*  *(-0.16,*  *0.01)* |
| **Smoking** | -6.10  (-11.16,  -1.04) | -7.33  (-12.42,  -1.04) | *-1.59*  *(-3.27,*  *0.09)* | -2.43  (-4.23,  -0.62) |  |  |  | *-0.38*  *(-0.79,*  *0.03)* |
| **EC status (ref=asymptomatic)** |  |  |  |  |  |  |  |  |
| **>50% in bed** | -22.70  (-32.73,  -12.68) |  | -14.67  (-18.00,  -11.34) |  | -8.37  (-11.77,  -4.97) |  | -2.40  (-3.18,  -1.62) |  |
| **<50% in bed** | -18.73  (-26.48,  -10.97) |  | -9.42  (-11.98,  -6.86) |  | -6.13  (-8.74,  -3.51) |  | -1.63  (-2.24,  -1.03) |  |
| **Sx, but**  **Ambulatory** | -7.86  (-14.32,  -1.39 |  | -3.42  (-5.53,  -1.31) |  | -1.70  (-3.86,  0.45) |  | -0.58  (-1.07,  -0.08) |  |
| **Education (ref=no high school diploma)** |  |  |  |  |  |  |  |  |
| **College (some or degree)** |  |  |  | *1.59 (-1.03,*  *4.22)* |  |  |  |  |
| **High school diploma** |  |  |  | *-0.10*  *(-2.77,*  *2.56)* |  |  |  |  |

Note: Parameter estimates represent the change in outcome for a 1 unit change in the independent variable, except for age which represents a 10-year change and MELD 3.0 which represents a 5-unit change. PROPr has been manipulated so that when interpreting parameter estimates (beta coefficients in the linear regression model), the change in response is .1 rather than 1 unit.

Detailed results including parameter estimates with 95%CI and p-values from linear regression modeling are provided below. **Yellow highlights in Tables 3-10 indicate significant relationships and blue highlights indicate borderline relationships. Table 11 provides F-statistics and p-values for model components across the outcomes.**

**Table 3: Model estimating FACT-Hep Total Score with ECOG**

| **Parameter** | **Estimate** | **Probt** | **Lower CL** | **Upper CL** |
| --- | --- | --- | --- | --- |
| **Intercept** | **102.00** | **<.0001** | **78.11** | **125.89** |
| **Female** | **1.57** | **0.5693** | **-3.86** | **7.01** |
| **White** | **-1.66** | **0.5429** | **-7.02** | **3.70** |
| **Age_10** | **7.28** | **<.0001** | **4.63** | **9.92** |
| **Insurance** | **-2.47** | **0.6141** | **-12.10** | **7.15** |
| **Education College (some or degree)** | **-1.64** | **0.6694** | **-9.18** | **5.90** |
| **Education High school diploma** | **-0.99** | **0.7980** | **-8.61** | **6.63** |
| **Education No high school diploma** | **0.00** | **.** | **.** | **.** |
| **Marital_Status Divorced** | **-3.15** | **0.2734** | **-8.78** | **2.49** |
| **Marital_Status Single** | **0.41** | **0.8864** | **-5.28** | **6.11** |
| **Marital_Status Widowed** | **0.40** | **0.9266** | **-8.04** | **8.83** |
| **Marital_Status Married** | **0.00** | **.** | **.** | **.** |
| **Ascites mild/mod (diur resp)** | **-4.67** | **0.0905** | **-10.09** | **0.74** |
| **Ascites severe (diur refr)** | **-6.23** | **0.0921** | **-13.47** | **1.02** |
| **Ascites none** | **0.00** | **.** | **.** | **.** |
| **Encephalopathy Grade 1-2** | **-1.13** | **0.6477** | **-6.00** | **3.74** |
| **Encephalopathy Grade 3-4** | **-2.79** | **0.5317** | **-11.53** | **5.96** |
| **Encephalopathy None** | **0.00** | **.** | **.** | **.** |
| **Hepatic cancer** | **1.87** | **0.5496** | **-4.26** | **8.00** |
| **ECOG Symptomatic, < 50% in bed during the day** | **-18.73** | **<.0001** | **-26.48** | **-10.97** |
| **ECOG Symptomatic, > 50% in bed during the day** | **-22.70** | **<.0001** | **-32.73** | **-12.68** |
| **ECOG Symptomatic, but completely ambulatory** | **-7.86** | **0.0173** | **-14.32** | **-1.39** |
| **ECOG Asymptomatic** | **0.00** | **.** | **.** | **.** |
| **CCI, no LD** | **-0.80** | **0.1332** | **-1.85** | **0.25** |
| **MELD_5** | **-2.88** | **0.0114** | **-5.10** | **-0.65** |
| **Current_smoker** | **-6.10** | **0.0183** | **-11.16** | **-1.04** |
| **Primary_Dis Autoimmune** | **3.64** | **0.6100** | **-10.37** | **17.65** |
| **Primary_Dis MASH** | **-1.24** | **0.6872** | **-7.27** | **4.80** |
| **Primary_Dis Other** | **0.62** | **0.8938** | **-8.56** | **9.81** |
| **Primary_Dis Viral** | **-1.07** | **0.7335** | **-7.23** | **5.10** |
| **Primary_Dis Alcohol related** | **0.00** | **.** | **.** | **.** |
| **BMI** | **-0.11** | **0.4751** | **-0.42** | **0.20** |

**Table 4: Model without ECOG (FACT-Hep total score)**

| **Parameter** | **Estimate** | **Probt** | **Lower CL** | **Upper CL** |
| --- | --- | --- | --- | --- |
| **Intercept** | **108.16** | **<.0001** | **85.44** | **130.88** |
| **Female** | **1.78** | **0.5189** | **-3.63** | **7.18** |
| **White** | **-1.97** | **0.4766** | **-7.39** | **3.46** |
| **Age_10** | **6.53** | **<.0001** | **3.93** | **9.12** |
| **Insurance** | **-0.95** | **0.8456** | **-10.49** | **8.60** |
| **Education College (some or degree)** | **-1.57** | **0.6782** | **-9.02** | **5.87** |
| **Education High school diploma** | **-1.77** | **0.6445** | **-9.31** | **5.76** |
| **Education No high school diploma** | **0.00** | **.** | **.** | **.** |
| **Marital_Status Divorced** | **-3.16** | **0.2750** | **-8.84** | **2.52** |
| **Marital_Status Single** | **-1.45** | **0.6169** | **-7.15** | **4.25** |
| **Marital_Status Widowed** | **0.50** | **0.9088** | **-7.99** | **8.98** |
| **Marital_Status Married** | **0.00** | **.** | **.** | **.** |
| **Ascites mild/mod (diur resp)** | **-4.86** | **0.0794** | **-10.30** | **0.57** |
| **Ascites severe (diur refr)** | **-8.19** | **0.0274** | **-15.47** | **-0.92** |
| **Ascites none** | **0.00** | **.** | **.** | **.** |
| **Encephalopathy Grade 1-2** | **-2.86** | **0.2457** | **-7.70** | **1.98** |
| **Encephalopathy Grade 3-4** | **-3.17** | **0.4773** | **-11.92** | **5.58** |
| **Encephalopathy None** | **0.00** | **.** | **.** | **.** |
| **Hepatic cancer** | **3.57** | **0.2599** | **-2.65** | **9.78** |
| **CCI, no LD** | **-0.97** | **0.0744** | **-2.03** | **0.10** |
| **MELD_5** | **-4.22** | **0.0001** | **-6.38** | **-2.06** |
| **Current_smoker** | **-7.33** | **0.0048** | **-12.42** | **-2.24** |
| **Primary_Dis Autoimmune** | **4.77** | **0.4981** | **-9.05** | **18.60** |
| **Primary_Dis MASH** | **-1.94** | **0.5321** | **-8.02** | **4.15** |
| **Primary_Dis Other** | **1.05** | **0.8227** | **-8.15** | **10.26** |
| **Primary_Dis Viral** | **-1.95** | **0.5391** | **-8.17** | **4.28** |
| **Primary_Dis Alcohol related** | **0.00** | **.** | **.** | **.** |
| **BMI** | **-0.29** | **0.0606** | **-0.59** | **0.01** |

**Table 5: Model with ECOG (PROMIS-29 Physical Health Score)**

| **Parameter** | **Estimate** | **Probt** | **Lower CL** | **Upper CL** |
| --- | --- | --- | --- | --- |
| **Intercept** | **45.67** | **<.0001** | **37.76** | **53.59** |
| **Female** | **-1.86** | **0.0422** | **-3.66** | **-0.07** |
| **White** | **1.18** | **0.1949** | **-0.60** | **2.96** |
| **Age_10** | **0.51** | **0.2532** | **-0.37** | **1.39** |
| **Insurance** | **2.37** | **0.1542** | **-0.89** | **5.64** |
| **Education College (some or degree)** | **2.00** | **0.1132** | **-0.48** | **4.48** |
| **Education High school diploma** | **0.94** | **0.4621** | **-1.57** | **3.45** |
| **Education No high school diploma** | **0.00** | **.** | **.** | **.** |
| **Marital_Status Divorced** | **0.42** | **0.6593** | **-1.45** | **2.29** |
| **Marital_Status Single** | **2.75** | **0.0045** | **0.86** | **4.64** |
| **Marital_Status Widowed** | **-0.16** | **0.9101** | **-2.89** | **2.58** |
| **Marital_Status Married** | **0.00** | **.** | **.** | **.** |
| **Ascites mild/mod (diur resp)** | **-2.43** | **0.0082** | **-4.22** | **-0.63** |
| **Ascites severe (diur refr)** | **-3.94** | **0.0014** | **-6.34** | **-1.53** |
| **Ascites none** | **0.00** | **.** | **.** | **.** |
| **Encephalopathy Grade 1-2** | **0.18** | **0.8244** | **-1.42** | **1.78** |
| **Encephalopathy Grade 3-4** | **0.92** | **0.5288** | **-1.94** | **3.78** |
| **Encephalopathy None** | **0.00** | **.** | **.** | **.** |
| **Hepatic cancer** | **-0.10** | **0.9234** | **-2.11** | **1.91** |
| **ECOG Symptomatic, but completely ambulatory** | **-3.42** | **0.0015** | **-5.53** | **-1.31** |
| **ECOG Symptomatic, gt 50% in bed during the day** | **-14.67** | **<.0001** | **-18.00** | **-11.34** |
| **ECOG Symptomatic, lt 50% in bed during the day** | **-9.42** | **<.0001** | **-11.98** | **-6.86** |
| **ECOG Asymptomatic** | **0.00** | **.** | **.** | **.** |
| **CCI, no LD** | **-0.18** | **0.2988** | **-0.53** | **0.16** |
| **meld_5** | **-0.33** | **0.3777** | **-1.06** | **0.40** |
| **Current_smoker** | **-1.59** | **0.0640** | **-3.27** | **0.09** |
| **Primary_Dis Autoimmune** | **0.48** | **0.8365** | **-4.10** | **5.06** |
| **Primary_Dis MASH** | **0.38** | **0.7057** | **-1.60** | **2.36** |
| **Primary_Dis Other** | **-1.08** | **0.4824** | **-4.12** | **1.95** |
| **Primary_Dis Viral (HBV, HCV)** | **-0.62** | **0.5482** | **-2.63** | **1.40** |
| **Primary_Dis Alcohol related** | **0.00** | **.** | **.** | **.** |
| **BMI** | **-0.15** | **0.0030** | **-0.26** | **-0.05** |

**Table 6: Model without ECOG (PROMIS-29 Physical Health Score)**

| **Parameter** | **Estimate** | **Probt** | **Lower CL** | **Upper CL** |
| --- | --- | --- | --- | --- |
| **Intercept** | **51.69** | **<.0001** | **43.60** | **59.78** |
| **Female** | **-2.07** | **0.0344** | **-3.98** | **-0.15** |
| **White** | **0.69** | **0.4827** | **-1.24** | **2.62** |
| **Age_10** | **-0.10** | **0.8370** | **-1.02** | **0.83** |
| **Insurance** | **2.51** | **0.1557** | **-0.96** | **5.99** |
| **Education College (some or degree)** | **1.59** | **0.2342** | **-1.03** | **4.22** |
| **Education High school diploma** | **-0.10** | **0.9402** | **-2.77** | **2.56** |
| **Education No high school diploma** | **0.00** | **.** | **.** | **.** |
| **Marital_Status Divorced** | **0.43** | **0.6788** | **-1.59** | **2.45** |
| **Marital_Status Single** | **1.89** | **0.0675** | **-0.14** | **3.91** |
| **Marital_Status Widowed** | **-0.10** | **0.9490** | **-3.04** | **2.85** |
| **Marital_Status Married** | **0.00** | **.** | **.** | **.** |
| **Ascites mild/mod (diur resp)** | **-2.67** | **0.0068** | **-4.60** | **-0.74** |
| **Ascites severe (diur refr)** | **-5.43** | **<.0001** | **-8.02** | **-2.85** |
| **Ascites none** | **0.00** | **.** | **.** | **.** |
| **Encephalopathy Grade 1-2** | **-0.54** | **0.5356** | **-2.24** | **1.16** |
| **Encephalopathy Grade 3-4** | **0.83** | **0.5966** | **-2.24** | **3.90** |
| **Encephalopathy None** | **0.00** | **.** | **.** | **.** |
| **Hepatic cancer** | **0.80** | **0.4745** | **-1.39** | **2.98** |
| **CCI, no LD** | **-0.29** | **0.1378** | **-0.67** | **0.09** |
| **MELD_5** | **-1.15** | **0.0033** | **-1.91** | **-0.38** |
| **Current_smoker** | **-2.43** | **0.0085** | **-4.23** | **-0.62** |
| **Primary_Dis Autoimmune** | **1.30** | **0.5988** | **-3.54** | **6.14** |
| **Primary_Dis MASH** | **0.25** | **0.8222** | **-1.90** | **2.39** |
| **Primary_Dis Other** | **-0.81** | **0.6238** | **-4.07** | **2.44** |
| **Primary_Dis Viral (HBV, HCV)** | **-0.87** | **0.4338** | **-3.05** | **1.31** |
| **Primary_Dis Alcohol related** | **0.00** | **.** | **.** | **.** |
| **BMI** | **-0.24** | **<.0001** | **-0.35** | **-0.14** |

**Table 7: Model with ECOG (PROMIS-29 Mental Health score)**

| **Parameter** | **Estimate** | **Probt** | **Lower CL** | **Upper CL** |
| --- | --- | --- | --- | --- |
| **Intercept** | **32.28** | **<.0001** | **24.19** | **40.37** |
| **Female** | **-0.25** | **0.7879** | **-2.09** | **1.58** |
| **White** | **-1.12** | **0.2281** | **-2.94** | **0.70** |
| **Age_10** | **3.04** | **<.0001** | **2.15** | **3.94** |
| **Insurance** | **-0.30** | **0.8582** | **-3.64** | **3.03** |
| **Education College (some or degree)** | **0.02** | **0.9863** | **-2.51** | **2.56** |
| **Education High school diploma** | **0.36** | **0.7836** | **-2.21** | **2.93** |
| **Education No high school diploma** | **0.00** | **.** | **.** | **.** |
| **Marital_Status Divorced** | **0.44** | **0.6501** | **-1.47** | **2.35** |
| **Marital_Status Single** | **2.88** | **0.0036** | **0.94** | **4.81** |
| **Marital_Status Widowed** | **0.08** | **0.9536** | **-2.71** | **2.88** |
| **Marital_Status Married** | **0.00** | **.** | **.** | **.** |
| **Ascites mild/mod (diur resp)** | **-0.74** | **0.4309** | **-2.57** | **1.10** |
| **Ascites severe (diur refr)** | **-1.91** | **0.1277** | **-4.37** | **0.55** |
| **Ascites none** | **0.00** | **.** | **.** | **.** |
| **Encephalopathy Grade 1-2** | **-1.04** | **0.2127** | **-2.67** | **0.60** |
| **Encephalopathy Grade 3-4** | **-0.86** | **0.5628** | **-3.79** | **2.06** |
| **Encephalopathy None** | **0.00** | **.** | **.** | **.** |
| **Hepatic cancer** | **0.54** | **0.6028** | **-1.51** | **2.60** |
| **ECOG Symptomatic, but completely ambulatory** | **-1.70** | **0.1213** | **-3.86** | **0.45** |
| **ECOG Symptomatic, less 50% in bed during the day** | **-6.13** | **<.0001** | **-8.74** | **-3.51** |
| **ECOG Symptomatic, more 50% in bed during the day** | **-8.37** | **<.0001** | **-11.77** | **-4.97** |
| **ECOG Asymptomatic** | **0.00** | **.** | **.** | **.** |
| **CCI, no LD** | **-0.38** | **0.0361** | **-0.74** | **-0.02** |
| **MELD_5** | **-0.57** | **0.1327** | **-1.32** | **0.17** |
| **Current_smoker** | **-0.73** | **0.4011** | **-2.45** | **0.98** |
| **Primary_Dis Autoimmune** | **2.07** | **0.3862** | **-2.61** | **6.75** |
| **Primary_Dis MASH** | **0.16** | **0.8731** | **-1.86** | **2.19** |
| **Primary_Dis Other** | **1.03** | **0.5130** | **-2.07** | **4.13** |
| **Primary_Dis Viral (HBV, HCV)** | **-0.88** | **0.4019** | **-2.94** | **1.18** |
| **Primary_Dis Alcohol related** | **0.00** | **.** | **.** | **.** |
| **BMI** | **-0.05** | **0.3382** | **-0.15** | **0.05** |

**Table 8: Model without ECOG (PROMIS-29 mental health score)**

| **Parameter** | **Estimate** | **Probt** | **Lower CL** | **Upper CL** |
| --- | --- | --- | --- | --- |
| **Intercept** | **35.32** | **<.0001** | **27.53** | **43.11** |
| **Female** | **-0.29** | **0.7590** | **-2.13** | **1.56** |
| **White** | **-1.41** | **0.1365** | **-3.27** | **0.45** |
| **Age_10** | **2.81** | **<.0001** | **1.92** | **3.70** |
| **Insurance** | **-0.09** | **0.9559** | **-3.44** | **3.25** |
| **Education College (some or degree)** | **0.05** | **0.9715** | **-2.48** | **2.58** |
| **Education High school diploma** | **-0.07** | **0.9548** | **-2.64** | **2.49** |
| **Education No high school diploma** | **0.00** | **.** | **.** | **.** |
| **Marital_Status Divorced** | **0.45** | **0.6505** | **-1.50** | **2.39** |
| **Marital_Status Single** | **2.29** | **0.0216** | **0.34** | **4.24** |
| **Marital_Status Widowed** | **0.16** | **0.9126** | **-2.68** | **3.00** |
| **Marital_Status Married** | **0.00** | **.** | **.** | **.** |
| **Ascites mild/mod (diur resp)** | **-0.88** | **0.3513** | **-2.74** | **0.98** |
| **Ascites severe (diur refr)** | **-2.79** | **0.0281** | **-5.28** | **-0.30** |
| **Ascites none** | **0.00** | **.** | **.** | **.** |
| **Encephalopathy Grade 1-2** | **-1.57** | **0.0600** | **-3.21** | **0.07** |
| **Encephalopathy Grade 3-4** | **-0.85** | **0.5723** | **-3.81** | **2.11** |
| **Encephalopathy None** | **0.00** | **.** | **.** | **.** |
| **Hepatic cancer** | **1.06** | **0.3244** | **-1.05** | **3.16** |
| **CCI, no LD** | **-0.46** | **0.0142** | **-0.82** | **-0.09** |
| **MELD_5** | **-1.03** | **0.0059** | **-1.77** | **-0.30** |
| **Current_smoker** | **-1.24** | **0.1623** | **-2.98** | **0.50** |
| **Primary_Dis Autoimmune** | **1.74** | **0.4628** | **-2.92** | **6.41** |
| **Primary_Dis MASH** | **0.02** | **0.9827** | **-2.04** | **2.08** |
| **Primary_Dis Other** | **0.86** | **0.5916** | **-2.28** | **3.99** |
| **Primary_Dis Viral (HBV, HCV)** | **-1.12** | **0.2947** | **-3.23** | **0.98** |
| **Primary_Dis Alcohol related** | **0.00** | **.** | **.** | **.** |
| **BMI** | **-0.11** | **0.0292** | **-0.22** | **-0.01** |

**Table 9: Model with ECOG (PROMIS-29 PROPr, reporting change in response as 0.1 unit)**

| **Parameter** | **Estimate** | **Probt** | **Lower CL** | **Upper CL** |
| --- | --- | --- | --- | --- |
| **Intercept** | **1.97** | **0.0387** | **0.10** | **3.83** |
| **Female** | **-0.20** | **0.3474** | **-0.63** | **0.22** |
| **White** | **-0.08** | **0.7159** | **-0.50** | **0.34** |
| **Age_10** | **0.42** | **<.0001** | **0.22** | **0.63** |
| **Insurance** | **0.10** | **0.8036** | **-0.67** | **0.87** |
| **Education College (some or degree)** | **0.08** | **0.7960** | **-0.51** | **0.66** |
| **Education High school diploma** | **0.13** | **0.6650** | **-0.46** | **0.72** |
| **Education No high school diploma** | **0.00** | **.** | **.** | **.** |
| **Marital_Status Divorced** | **0.10** | **0.6408** | **-0.34** | **0.54** |
| **Marital_Status Single** | **0.75** | **0.0010** | **0.31** | **1.19** |
| **Marital_Status Widowed** | **0.02** | **0.9474** | **-0.62** | **0.67** |
| **Marital_Status Married** | **0.00** | **.** | **.** | **.** |
| **Ascites mild/mod (diur resp)** | **-0.33** | **0.1278** | **-0.75** | **0.09** |
| **Ascites severe (diur refr)** | **-0.59** | **0.0427** | **-1.15** | **-0.02** |
| **Ascites none** | **0.00** | **.** | **.** | **.** |
| **Encephalopathy Grade 1-2** | **-0.18** | **0.3454** | **-0.56** | **0.20** |
| **Encephalopathy Grade 3-4** | **-0.18** | **0.5902** | **-0.86** | **0.49** |
| **Encephalopathy None** | **0.00** | **.** | **.** | **.** |
| **Hepatic cancer** | **0.14** | **0.5724** | **-0.34** | **0.61** |
| **ECOG Symptomatic, but completely ambulatory** | **-0.58** | **0.0230** | **-1.07** | **-0.08** |
| **ECOG Symptomatic, gt 50% in bed during the day** | **-2.40** | **<.0001** | **-3.18** | **-1.62** |
| **ECOG Symptomatic, lt 50% in bed during the day** | **-1.63** | **<.0001** | **-2.24** | **-1.03** |
| **ECOG Asymptomatic** | **0.00** | **.** | **.** | **.** |
| **CCI, no LD** | **-0.06** | **0.1450** | **-0.14** | **0.02** |
| **MELD_5** | **-0.10** | **0.2609** | **-0.27** | **0.07** |
| **Current_smoker** | **-0.25** | **0.2205** | **-0.64** | **0.15** |
| **Primary_Dis Autoimmune** | **0.26** | **0.6348** | **-0.82** | **1.34** |
| **Primary_Dis MASH** | **0.06** | **0.7857** | **-0.40** | **0.53** |
| **Primary_Dis Other** | **0.08** | **0.8240** | **-0.63** | **0.79** |
| **Primary_Dis Viral (HBV, HCV)** | **-0.19** | **0.4268** | **-0.67** | **0.28** |
| **Primary_Dis Alcohol related** | **0.00** | **.** | **.** | **.** |
| **BMI** | **-0.02** | **0.0802** | **-0.05** | **0.00** |

**Table 10: Model without ECOG (PROMIS-29 PROPr, parameter estimates for a 0.1 unit change in response)**

| **Parameter** | **Estimate** | **Probt** | **Lower CL** | **Upper CL** |
| --- | --- | --- | --- | --- |
| **Intercept** | **2.82** | **0.0025** | **1.00** | **4.64** |
| **Female** | **-0.22** | **0.3113** | **-0.65** | **0.21** |
| **White** | **-0.13** | **0.5444** | **-0.57** | **0.30** |
| **Age_10** | **0.35** | **0.0011** | **0.14** | **0.55** |
| **Insurance** | **0.14** | **0.7315** | **-0.64** | **0.92** |
| **Education College (some or degree)** | **0.11** | **0.7124** | **-0.48** | **0.70** |
| **Education High school diploma** | **0.05** | **0.8642** | **-0.55** | **0.65** |
| **Education No high school diploma** | **0.00** | **.** | **.** | **.** |
| **Marital_Status Divorced** | **0.10** | **0.6573** | **-0.35** | **0.56** |
| **Marital_Status Single** | **0.59** | **0.0114** | **0.13** | **1.04** |
| **Marital_Status Widowed** | **0.04** | **0.9160** | **-0.63** | **0.70** |
| **Marital_Status Married** | **0.00** | **.** | **.** | **.** |
| **Ascites mild/mod (diur resp)** | **-0.36** | **0.1038** | **-0.79** | **0.07** |
| **Ascites severe (diur refr)** | **-0.82** | **0.0057** | **-1.40** | **-0.24** |
| **Ascites none** | **0.00** | **.** | **.** | **.** |
| **Encephalopathy Grade 1-2** | **-0.33** | **0.0956** | **-0.71** | **0.06** |
| **Encephalopathy Grade 3-4** | **-0.20** | **0.5692** | **-0.89** | **0.49** |
| **Encephalopathy None** | **0.00** | **.** | **.** | **.** |
| **Hepatic cancer** | **0.27** | **0.2788** | **-0.22** | **0.76** |
| **CCI, no LD** | **-0.08** | **0.0679** | **-0.16** | **0.01** |
| **MELD_5** | **-0.24** | **0.0066** | **-0.41** | **-0.07** |
| **Current_smoker** | **-0.38** | **0.0670** | **-0.79** | **0.03** |
| **Primary_Dis Autoimmune** | **0.26** | **0.6366** | **-0.83** | **1.35** |
| **Primary_Dis MASH** | **0.03** | **0.9057** | **-0.45** | **0.51** |
| **Primary_Dis Other** | **0.09** | **0.8147** | **-0.65** | **0.82** |
| **Primary_Dis Viral (HBV, HCV)** | **-0.27** | **0.2834** | **-0.76** | **0.22** |
| **Primary_Dis Alcohol related** | **0.00** | **.** | **.** | **.** |
| **BMI** | **-0.04** | **0.0013** | **-0.06** | **-0.02** |

**Table 11: F-statistics and p-values for the components of the full models estimating FACT-Hep, PROMIS Physical Health, PROMIS Mental Health, and PROPr**

|  | FACT-Hep Total | | PROMIS Physical Health | | PROMIS Mental Health | | PROPr Summary | |
| --- | --- | --- | --- | --- | --- | --- | --- | --- |
|  | With EC status | Without EC status | With EC status | Without EC status | with EC status | Without EC status | with EC status | Without EC status |
| Gender | 0.32 (0.57) | 0.42 (0.52) | 4.15 (.042) | 4.50 (0.034) | 0.07 (0.79) | 0.09 (0.76) | 0.88 (0.35) | 1.03 (0.31) |
| Race | 0.37 (0.54) | 0.51 (0.48) | 1.68 (0.19) | 0.49 (0.48) | 1.46 (0.23) | 2.22 (0.14) | 0.13 (0.72) | 0.37 (0.54) |
| Age | 29.26 (<0.001) | 24.38 (<0.001) | 1.31 (0.25) | 0.04 (0.84) | 44.65 (<0.001) | 38.62 (<0.001) | 16.32 (<0.001) | 10.81 (0.001) |
| Insurance | 0.25 (0.61) | 0.04 (0.85) | 2.04 (0.14) | 2.02 (0.16) | 0.03 (0.86) | 0.0 (0.96) | 0.06 (0,80) | 0.12 (0.73) |
| Education, 2df | 0.10 (0.90) | 0.11 (0.90) | 1.75 (0.18) | 2.33 (0.099) | 0.10 (0.90) | 0.01 (0.99) | 0.11 (0.90) | 0.09 (0.91) |
| Marital status, 3df | 0,52 (0.67) | 0.44 (0.72) | 2.91 (0.034) | 1.17 (0.32) | 3.00 (0.030) | 1.82 (0.14) | 3.87 (0.009) | 2.23 (0.0832) |
| Ascites, 2df | 1.85 (0.16) | 2.65 (0.071) | 5.71 (0.004) | 8.64 (<0.001) | 1.17 (0.31) | 2.49 (0.08) | 2.18 (0.11) | 3.85 (0.0218) |
| HE, 2df | 0.24 (0.79)) | 0.75 (0.47) | 0.20 (0.82) | 0.47 (.63) | 0.80 (0.45) | 1.78 (0.17) | 0.48 (0.62) | 1.40 (0.25) |
| HCC | 0.36 (0.55) | 1.27 (0.26) | 0.01 (.92) | 0.51 (0.47) | 0.27 (0.60) | 0.97 (0.32) | 0.32 (0.57) | 1.18 (0.28) |
| CCI (no age or lv dz) | 2.26 (0.13) | 3.19 (0.074) | 1.08 (0.30) | 2.21 (0.14) | 4.42 (0.036) | 6.05 (0.014) | 2.13 (0.15) | 3.35 (0.068) |
| MELD | 6.44 (0.011) | 14.72 (<0.001) | 0.78 (0.38) | 8.72 (0.003) | 2.27 (0.13) | 7.64 (0.006) | 1.27 (0.26) | 7.43 (0.007) |
| Smoking | 5.61 (0.018) | 8.01 (0.005) | 3.44 (0.064) | 6.98 (0.009) | 0.71 (.40) | 1.96 (0.16) | 1.50 (0.22) | 3.37 (0.067) |
| Liver etiology, 4df | 0.16 (0.96) | 0.36 (0.84) | 0.35 (0.84) | 0.36 (0.84) | 0.59 (0.67)) | 0.61 (0.65) | 0.33 (0.85) | 0.47 (0.76) |
| BMI | 0.51 (0.48) | 3.53 (0.061) | 8.91 (0.003) | 20.45 (<0.001) | 0.92 (0.34) | 4.78 (0.029) | 3.07 (0.08) | 10.51 (0.001) |
| ECOG, 3 df | 11.11 (<.001) |  | 36.73 (<0.001) |  | 14.15 (<0.001) |  | 18.70 (<0.001) |  |
